# Cross-reactive antibodies against Langat virus protect mice from lethal tick-borne encephalitis virus infection

**DOI:** 10.3389/fimmu.2023.1134371

**Published:** 2023-02-28

**Authors:** Mareike Kubinski, Jana Beicht, Isabel Zdora, Giulietta Saletti, Magdalena Kircher, Monique Petry-Gusmag, Imke Steffen, Christina Puff, Klaus Jung, Wolfgang Baumgärtner, Guus F. Rimmelzwaan, Albert D. M. E. Osterhaus, Chittappen Kandiyil Prajeeth

**Affiliations:** ^1^ Research Center for Emerging Infections and Zoonoses, University of Veterinary Medicine Hannover, Foundation, Hannover, Germany; ^2^ Department of Pathology, University of Veterinary Medicine Hannover, Foundation, Hannover, Germany; ^3^ Center of Systems Neuroscience, Hannover Graduate School for Neurosciences, Infection Medicine, and Veterinary Sciences (HGNI), Hannover, Germany; ^4^ Institute for Animal Breeding and Genetics, University of Veterinary Medicine Hannover, Foundation, Hannover, Germany; ^5^ Institute for Biochemistry, University of Veterinary Medicine Hannover, Foundation, Hannover, Germany

**Keywords:** TBEV, immunity, cross-reactivity, LGTV, CNS

## Abstract

**Introduction:**

Naturally attenuated Langat virus (LGTV) and highly pathogenic tick-borne encephalitis virus (TBEV) share antigenically similar viral proteins and are grouped together in the same flavivirus serocomplex. In the early 1970s, this has encouraged the usage of LGTV as a potential live attenuated vaccine against tick-borne encephalitis (TBE) until cases of encephalitis were reported among vaccinees. Previously, we have shown in a mouse model that immunity induced against LGTV protects mice against lethal TBEV challenge infection. However, the immune correlates of this protection have not been studied.

**Methods:**

We used the strategy of adoptive transfer of either serum or T cells from LGTV infected mice into naïve recipient mice and challenged them with lethal dose of TBEV.

**Results:**

We show that mouse infection with LGTV induced both cross-reactive antibodies and T cells against TBEV. To identify correlates of protection, Monitoring the disease progression in these mice for 16 days post infection, showed that serum from LGTV infected mice efficiently protected from developing severe disease. On the other hand, adoptive transfer of T cells from LGTV infected mice failed to provide protection. Histopathological investigation of infected brains suggested a possible role of microglia and T cells in inflammatory processes within the brain.

**Discussion:**

Our data provide key information regarding the immune correlates of protection induced by LGTV infection of mice which may help design better vaccines against TBEV.

## 1 Introduction

Flaviviruses are closely related RNA viruses that are capable of causing a spectrum of life-threatening diseases in humans. These include arthropod-borne disease-causing viruses such as tick-borne encephalitis virus (TBEV), dengue virus (DENV), Zika virus (ZIKV), Japanese encephalitis virus (JEV), yellow fever virus (YFV) and West Nile virus (WNV) (1). The single positive-stranded genomic RNA of flaviviruses encodes a single polyprotein which is cleaved into three structural proteins (capsid (C), envelope (E) and membrane (M)) and seven non-structural proteins (NS1, NS2A, NS2B, NS3, NS4A, NS4B and NS5) (2). Natural infection and vaccination with currently licensed inactivated whole TBEV vaccines evoke potent host immune responses against viral antigens. Interestingly, immune responses against flaviviruses were shown to cross-react with closely and distantly related viruses within this family (1, 3, 4). Cross-reactive immunity can either be protective or may contribute to disease enhancement (5–8). Serological analysis has identified antigenically related flaviviruses and grouped them into distinct serocomplexes (9). For instance, sera from individuals vaccinated against TBEV were shown to neutralize naturally attenuated Langat virus (LGTV), Kyasanur forest disease virus, Alkhurma virus and Powassan virus reminiscent of their grouping into the TBEV serocomplex (3). TBEV and LGTV share greater than 80% amino acid identity in the E protein (10). In humans, TBEV infections are mostly asymptomatic or lead to mild symptoms such as fever and headache. However, after several weeks, some patients develop tick-borne encephalitis (TBE) which may be life-threatening and lead to lifelong disabilities. In the early 1970s, LGTV was used as a candidate live-attenuated vaccine against TBEV infection in certain highly endemic areas of Russia. This was considered highly effective until encephalitis incidence of about 1:10000 was reported among vaccinees (10, 11). Consequently, the use of LGTV-based live vaccines has been discontinued. Currently, two formalin-inactivated whole TBEV vaccines (European strains) are used in Europe (12, 13). For effective prevention of TBE, manufacturers recommend administration of booster doses at three to five year intervals. However, data presented in a recent report suggests that these vaccines are still effective even when the booster intervals are extended to 10 years (14). Nevertheless, vaccine failures and breakthroughs have been reported in endemic areas and is a serious matter of concern (14–18). In contrast, the ability of live viral vaccines to induce both humoral- and cell-mediated immune responses, is highly appreciated. To further explore the potential of future candidate live attenuated TBE vaccines, a better understanding of the cross-immunity against TBEV induced by LGTV infection is important. In the present study, we dissected the contributions of cross-reactive antibodies and T cells in providing protection against lethal TBEV infection by adoptively transferring either serum or T cells from LGTV infected donor mice into naïve recipient mice. To this end, we have monitored disease progression, virus replication and histopathology in organs of mice challenged with TBEV upon LGTV infection.

## 2 Material and methods

### 2.1 Ethical statement

All animal experiments were conducted in strict compliance with European guidelines (EU directive on animal testing 2010/63/EU) and German Animal Welfare Law. The study protocol was approved by the Lower Saxony State Office for Consumer Protection and Food Safety (approval no. 33.8-42502-04-19/3259).

### 2.2 Mice

Female, 4-10 weeks old C57BL/6JOlaHsd (BL6) mice were purchased from Envigo RMS GmbH and were housed under pathogen-free conditions at the animal facility of the University of Veterinary Medicine Hannover, Foundation, in individually ventilated cages type Sealsafe Plus GM500 or IsoCage N Biocontainment system (Tecniplast) for the entire duration of the experiment. All mice were subjected to two weeks of habituation and acclimatization before they were taken into the experiments. Sterilized food pellets and water were provided *ad libitum*.

### 2.3 Viruses and cell culture

LGTV strain TP21 was obtained from Helmholtz Centre for Infection Research, Department of Molecular Immunology, Brunswick, Germany. Viral stocks were generated in VeroE6 cells and infectious virus titer was determined according to method described by Reed and Muench and expressed as tissue culture infectious dose 50% (TCID50) (19). TBEV strain Neudoerfl was provided by the Department of Microbiology of the German Armed Forces, Munich, Germany. Virus propagation and determination of titer by TCID50 assay were done in A549 cells. VeroE6 cells were grown in Eagle’s minimum essential medium (EMEM, Sigma-Aldrich) supplemented with 10% fetal bovine serum (FBS), 1% penicillin/streptomycin (Pen/Strep), 1% GlutaMAX™ and 20 mM HEPES. A549 cells were cultured in F-12 Nut Mix (1X) + GlutaMAX-I (Gibco™) containing 10% FBS, 1% Pen/Strep, 1% GlutaMAX™ and 20 mM HEPES. Cells were stored at 37°C/5% CO_2_. All cell lines and viral stocks were tested negative for mycoplasma.

### 2.4 LGTV immunogenicity study

Six- to eight-week-old BL6 mice (n=6 per group) were inoculated subcutaneously (s.c.) with 1.4x10^4^ TCID50 LGTV TP21 in 100 µl PBS or PBS only. At 28 days post infection (dpi), mice were bled by retrobulbar sinus puncture under isoflurane induced anesthesia and blood was collected in MiniCollect^®^ CAT Serum Sep Clot Activator tubes (Greiner Bio-One GmbH). To obtain sera, blood was kept for 30 min at room temperature (RT) and centrifuged at 3000x*g* for 10 min. Subsequently, mice were euthanized by cervical dislocation and spleens were collected. Single-cell suspensions from mouse spleens were prepared by using cell strainers and erythrocyte lysis was performed using ACK Lysing buffer (Gibco™). Subsequently, cells were resuspended in RPMI 1640 (1X) (Gibco™) + 10% FBS + 1% Pen/Strep + 5 mM ß-mercaptoethanol (R10F) for further use.

### 2.5 Adoptive transfer experiments

Donor mice (n=5 per group) were immunized by administering PBS or LGTV as described above. At 28 dpi, sera and spleens were collected from these mice. Sera from respective groups were pooled before transferring into naïve recipient mice. Similarly, splenocytes from each group were pooled and CD3^+^ T cells were isolated using the autoMACS^®^ Pro Separator (Miltenyi Biotec B.V. & Co. KG) with the mouse Pan T Cell Isolation Kit II (Miltenyi Biotec B.V. & Co. KG). Ten to twelve weeks old naïve recipient mice (n=5 per group) either received 200 µl of serum or 2.53x10^7^ CD3^+^ T cells intraperitoneally (i.p.). After 4 h, recipient mice were challenged s.c. with 5.4x10^3^ TCID50 TBEV Neudoerfl (100 µl). Following TBEV challenge infection, mice were monitored daily for a period of 16 dpi. Mice that developed clinical signs were given certain scores as per the information provided in the scoring sheet (**Supplementary Table S1**). Based on the clinical score attained, the humane endpoint (HEP) was determined and those mice that reached HEP were sacrificed. All other mice were monitored until 16 dpi (study endpoint) and subsequently euthanized. At sacrifice, serum was collected as described above. Left hemisphere of the brain, spinal cord and spleen were collected in PBS, homogenized with a stainless-steel bead by using the TissueLyser II (Qiagen) with 30 Hz for 1 min and stored at -80°C.

### 2.6 Virus neutralization assay

LGTV and TBEV virus neutralizing antibody titers (VNT) in serum of control and LGTV infected mice were determined by using virus neutralization assay (VNA) on VeroE6 and A549 cells, respectively, with 80% confluence. Sera was heat-inactivated for 30 min at 56°C and 2-fold serial dilutions were prepared in infection medium (same as growth medium but with 2% FBS). Serum dilutions were mixed with 100 TCID50 of LGTV TP21 or TBEV Neudoerfl and incubated for 1 h at 37°C/5% CO_2_. Serum-virus mix was added to VeroE6 or A549 cells and incubated at 37°C/5% CO_2_. Read-out based on presence/absence of cytopathic effect (CPE) was done after 5-6 days. VNT100 was determined as the reciprocal of the highest serum dilution where no CPE was visible.

### 2.7 Luciferase immunoprecipitation systems assay

Luciferase immunoprecipitation systems (LIPS) assay was performed as described previously (20). Briefly, supernatants containing TBEV-specific fusion proteins (C, prM, E-DIII, NS1, NS3-DIII, NS4b) or fusion protein without insert (secNLuc, control) were incubated with 1:100 dilution of heat-inactivated mouse serum. Luminescence was measured using the microplate reader infinite 200Pro (Tecan) with Tecan i-control software (version 2.0.10.0, Tecan). Average of triplicate measurements was determined and data was expressed as log_10_ relative light units (RLU). Luminescence values higher than the average of negative samples plus five-times the standard deviation are considered positive.

### 2.8 *Ex vivo* restimulation of splenocytes

Short peptide oligomers (15-mers with 11 amino acid overlaps) spanning the entire C, E, NS1, NS3 and NS5 proteins of TBEV Neudoerfl (UniProtKB: P14336) were synthesized (≥ 75% purity, GenScript Biotech Corp). Lyophilized peptides were reconstituted in DMSO (Hybri-Max™, Sigma-Aldrich) and peptide pools were generated as described in **Supplementary Table S2**. Concentration of each peptide in the pool was adjusted to 10 µg/ml and was used at final concentration of 1 µg/ml. Splenocytes (0.5-1x10^6^ cells/well) were restimulated with respective peptide pools. Negative controls were treated with DMSO or R10F. Positive controls were treated with mixture of 30 ng/ml Phorbol 12-myrisate 13-acetate (PMA; Cayman Chemical) and 0.5 µg/ml Ionomycin (Cayman Chemical).

### 2.9 IFN-γ ELISpot assay

Splenocytes (5x10^5^ splenocytes/well) restimulated as described above were tested for IFN-γ producing cells using mouse IFN-γ ELISpot Plus kit (Mabtech). For positive control (PMA/ionomycin stimulation), only 5x10^4^ splenocytes/well were used. The assay was carried out according to manufacturer’s instructions. Following overnight incubation with respective peptide pools at 37°C/5% CO_2_, plates were stained, developed and scanned using the ImmunoSpot^®^ S6 Ultimate Reader (Cellular Technology Limited). ImmunoSpot^®^ software (version 7.0.20.1, Cellular Technology Limited) was used for counting spots and data analysis. IFN-γ spots per 10^6^ splenocytes were calculated and duplicate measurements were averaged. After subtraction of negative control, data were shown as IFN-γ spot-forming cells (SFC)/10^6^ splenocytes.

### 2.10 Flow cytometry analysis

Following restimulation of splenocytes with respective peptide pools (as described above) for 6 h at 37°C/5% CO_2,_ T cells were further characterized by performing intracellular cytokine staining and flow cytometry. To block cytokine secretion from activated T cells, Brefeldin A (10 µg/ml, Sigma-Aldrich) was added to the medium for the final 4 h of restimulation. Cells were stained with LIVE/DEAD™ Fixable Near-IR Dead Cell Stain Kit for 633 or 635 nm excitation (Invitrogen™) for 20 min in the dark. Fc blocking was done with anti-Mouse CD16-CD32 (Clone: 93) for 15 min at RT. Surface staining using anti-Ms CD3e FITC (Clone: 145-2C11), anti-Ms CD4 PE (Clone RM4-5) and anti-Ms CD8a PerCP-Cyanine5.5 (Clone: 53-6.7) was done for 20 min at 4°C in the dark. After fixation and permeabilization with BD Cytofix/Cytoperm™ (BD Biosciences) for 20 min at 4°C in the dark, intracellular staining using anti-Ms IFN- γ APC (Clone: XMG1.2) and anti-hu/ms Granzyme B BV421 (Clone: QA18A28, BioLegend^®^) was performed for 30 min at 4°C in the dark. Finally, cells were resuspended in PBS and acquired by BD LSR Fortessa X-20 (BD Biosciences) using BD FACSDiva (version 9.0, BD Biosciences). All antibodies were purchased from eBioSciences™ (Invitrogen™) unless otherwise stated. Data analysis was performed by FlowJo™ software (version 10.8.1, BD Biosciences).

### 2.11 Determination of infectious virus titers

A 1:10 serial dilution of serum or organ homogenate (free of cell debris) was prepared in A549 infection medium (same as growth medium but with only 2% FBS) and transferred to approximately 80% confluent A549 cells. After 5-6 days at 37°C/5% CO_2_, TCID50 values for individual samples were determined by CPE-based read-out as described above. Detection limit for each organ titration was defined as lowest homogenate dilution (10^1^) divided by the average of respective organ weights.

### 2.12 RNA isolation and real time quantitative reverse transcription PCR

Total RNA of serum (pre-diluted 1:10 in A549 medium) or organ homogenate (free of cell debris) was isolated using QIAmp^®^ Viral RNA Mini Kit (Qiagen) according to manufacturer’s manual. For detection of TBEV RNA, real time quantitative reverse transcription (RT)-PCR using One-Step RT-PCR Kit (Qiagen) was performed based on the protocol established by Schwaiger and Cassinotti (21) with few modifications. To determine TBEV RNA copies, a dilution row of TBEV Neudoerfl RNA standard was used. The standard was kindly provided by Stefanie Becker (Institute for Parasitology and Research Center for Emerging Infections and Zoonoses at University of Veterinary Medicine Hannover, Foundation). As negative control, AVE buffer instead of sample was used. Real time quantitative RT-PCR was performed in duplicates using AriaMx Real-time PCR System (Agilent Technologies) with Agilent Aria software (version 1.5, Agilent Technologies). Cq values were converted into log_10_ TBEV copies/ml or gram tissue, respectively, according to the standard curve.

### 2.13 Histology

For histopathological analysis, the right hemisphere of the brain and the gastrointestinal tract were collected and fixed in ROTI^®^Histofix 4% (4% formaldehyde, Roth) for a minimum of 48 h. Two longitudinal sections of the brain and representative sections of duodenum, jejunum, ileum, caecum, colon and rectum of all mice were embedded in paraffin wax followed by cutting 2-3 µm thick sections using a microtome. Sections were stained with hematoxylin and eosin (H&E).

### 2.14 Histological evaluation

H&E stained sections of brain and intestine were analyzed using a semiquantitative scoring system. Ten different regions of the brain including olfactory bulb, cerebral cortex, basal forebrain, hippocampus, thalamus, hypothalamus, midbrain, pons, medulla and cerebellum were investigated applying six scoring categories. Scores were generated for each evaluable brain region separately. Meninges, perivascular as well as vascular inflammation, vascular lesions including perivascular edema, hemorrhage and fibrinoid necrosis, microgliosis characterized by hyperplasia and/or hypertrophy of microglia/macrophages as well as cellular necrosis characterized by karyorrhexis, karyolysis, pyknosis and triangularly shaped, hyperosinophilic and shrunken neurons were evaluated using a scoring system detailed in **Supplementary Table S3**. Duodenum, jejunum, ileum, caecum, colon and rectum were scored using five different categories. Hypercellularity/inflammatory infiltrates within the *lamina propria* of the *tunica mucosa* were evaluated. Additionally, *plexus submucosus* and *plexus myentericus* ganglia were each scored regarding necrosis of ganglion neurons characterized by karyorrhexis, karyolysis and pyknosis, hyperosinophilia and shrinkage of neurons as well as inflammatory infiltrates/hypercellularity (for detailed scoring system see **Supplementary Table S4**).

### 2.15 Immunohistochemistry

Immunohistochemistry (IHC) was performed as described previously (22) applying the avidin-biotin-peroxidase (ABC) complex method and using antibodies for the detection of TBEV (anti-TBEV E protein clone 1493, Matthias Niedrig, mouse monoclonal), T cells (anti-CD3, Agilent Dako, Cat.No. A0452, rabbit polyclonal), B-lymphocytes (anti-CD45R, BD Bioscience, Cat.No. 553085, rat monoclonal), microglia/macrophages (anti-ionized calcium-binding adapter molecule 1 (Iba1), Wako Chemicals, 019-19741, polyclonal rabbit) and astrocytes (anti-glial fibrillary acidic protein (GFAP), Dako Cytomation, Cat.No. Z0334, rabbit polyclonal). Briefly, sections of brain and intestine were dewaxed and rehydrated in a graded series of alcohol. For anti-CD3, anti-CD45R and anti-Iba1 antibodies, antigen retrieval was achieved by boiling sections in citrate buffer (pH = 6) in a microwave (800W) prior to blocking of unspecific binding sites. For anti-TBEV and anti-GFAP antibodies, no pretreatment was necessary. After overnight incubation of primary antibodies, sections were incubated with the respective biotinylated secondary antibodies for 45 min. The staining was visualized using chromogen 3,3’-diaminobenzidine tetrahydrochloride (DAB) and counterstaining of nuclei with Mayer’s hematoxylin (Roth C.GmbH & Co KG).

### 2.16 Immunohistochemical evaluation

The immunohistochemical stains of brain and intestine with TBEV, CD3, CD45R, Iba1 and GFAP were analyzed using a semiquantitative scoring scheme. No infiltration with CD45R-positive B-lymphocytes and no altered staining for GFAP were evident and therefore not included in further analyses. Detection and distribution of TBEV-positive cells, CD3-positive T cells, and Iba1-positive microglia/macrophages in the brain and intestinal tissue sections were evaluated using the scoring system detailed in **Supplementary Table S5**.

### 2.17 Statistical analysis

The immunogenicity and survival data were analyzed using GraphPad Prism software (version 9.0.0, GraphPad Software Inc.). For comparison of VNT100, LIPS and ELISpot data unpaired t-test was used. Survival data are shown as Kaplan-Meier curves and were analyzed by log rank test. Histopathological and IHC scorings were analyzed using R (version 4.2, www.r-project.org) and described using mean (median, minimum, maximum) per experimental group. The effects of transfer group (CD3^+^, Serum), treatment (control, LGTV) as well as the interaction of group and treatment were first analyzed using non-parametric ANOVA (23). In case of significant effects in the ANOVA, subsequent pairwise comparisons between experimental groups were performed using Wilcoxon rank sum tests. Raw p-values from the Wilcoxon tests were adjusted for multiple testing within sets of scores related to the same organ region using the method of Bonferroni-Holm. A *p-value <*0.05 was considered significant

## 3 Results

### 3.1 Infection of mice with LGTV induces cross-reactive antibodies against TBEV

**Figure 1:**
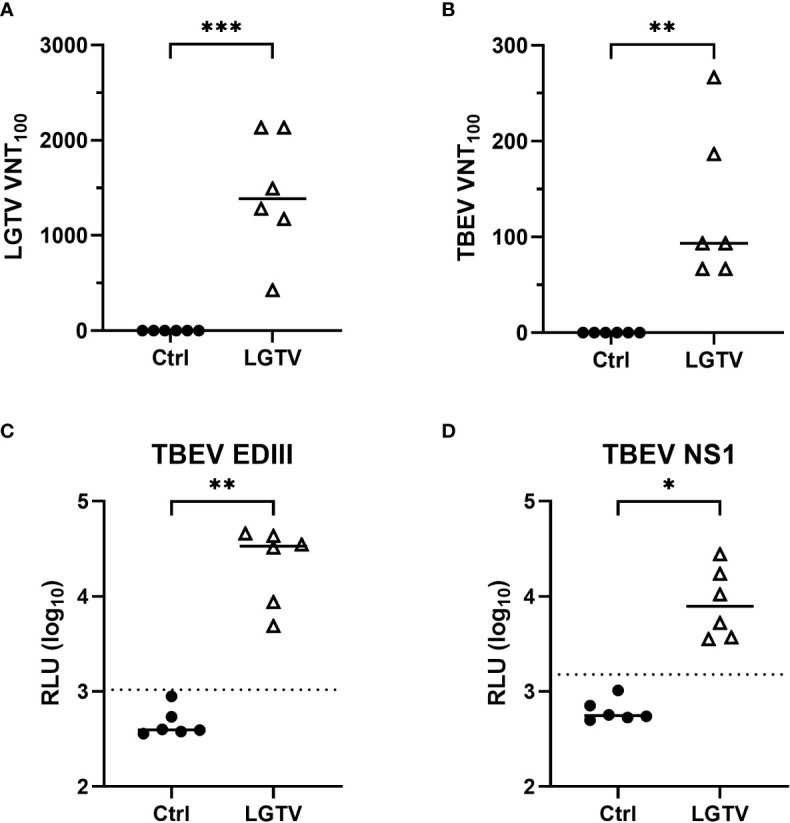
TBEV cross-reactive antibodies in the serum of LGTV immunized mice. Serum collected from control (•) or LGTV (Δ) infected mice (n=6) were tested for their ability to block the infection of **(A)** LGTV (p = 0.0003) and **(B)** TBEV (p = 0.0029) in VeroE6 and A549 cells, respectively. The graphs show the titer at which 100% virus neutralization is achieved (VNT_100_). LIPS assay with 1:100 dilution of serum detected antibodies against **(C)** domain III of the TBEV E protein (p = 0.0029) and **(D)** TBEV NS1 protein (p = 0.0214). Luciferase activity was measured in three independent experiments and is displayed as mean values of log_10_ RLU. Luminescence values higher than the average of negative samples plus five-times the standard deviation (dotted line) are considered positive. Median is shown in all graphs. *p<0.05, **p<0.01 and ***p<0.001.

Previously we have shown that s.c. administration of LGTV protects mice against subsequent lethal challenge infection with TBEV (24). In the present study, we aimed at defining the correlates of protection induced by LGTV acting against TBEV. Sera collected from control and LGTV infected mice at 28 dpi were tested for the presence of virus neutralizing (VN) antibodies against LGTV and TBEV. As expected, high titers of LGTV-neutralizing antibodies were observed in LGTV infected mice (**Figure 1A**). Interestingly, the same sera also displayed considerable neutralizing activity against TBEV, although the VN titers were 10-fold lower than those to LGTV (**Figure 1B**). Using LIPS assay, we could demonstrate the presence of cross-reactive antibodies against domain III of TBEV E and NS1 proteins (**Figures 1C, D**).

### 3.2 LGTV specific T cells cross-react with TBEV antigens

**Figure 2:**
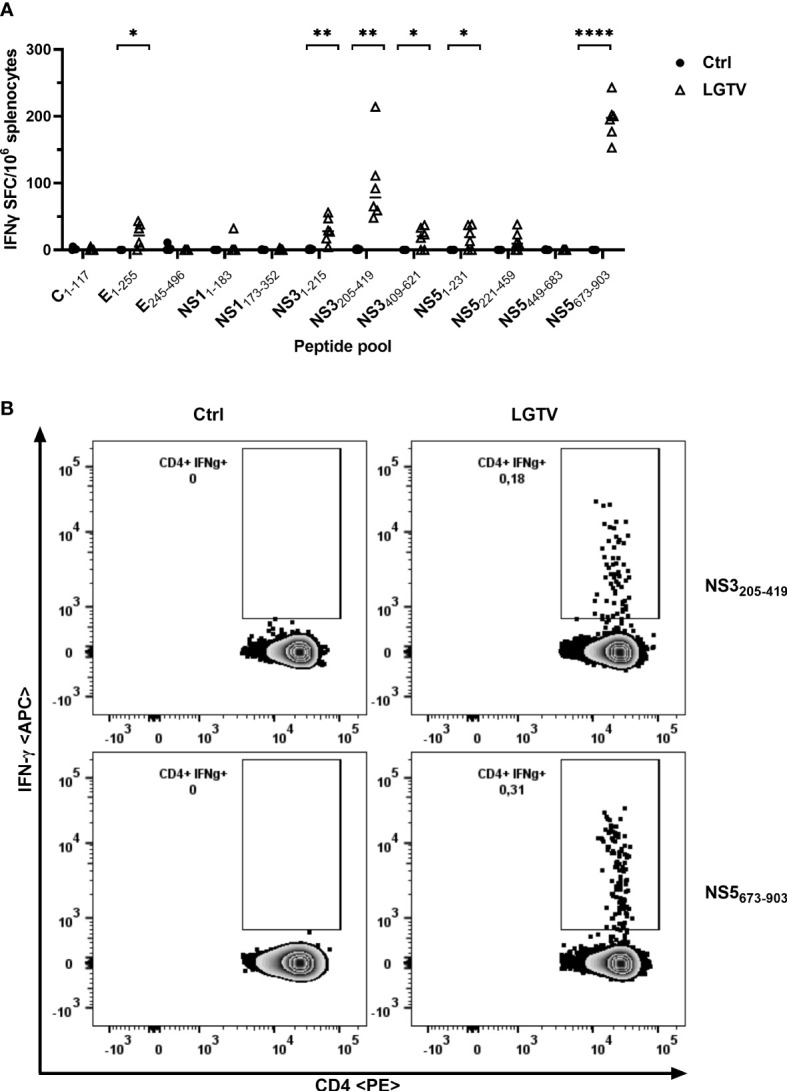
T cells induced by LGTV cross-react with TBEV antigenic peptides. **(A)** Splenocytes collected from control (•) or LGTV (Δ) infected mice (n=6) were restimulated with TBEV-specific peptide pools and the frequency of IFN-γ producing cells were determined using IFN-γ ELISpot assay. For statistical analysis, unpaired t-test was used (E_1-255_: p = 0.0168; NS3_1-215_: p = 0.0038; NS3_205-419_: p = 0.003; NS3_409-621_: p = 0.0178; NS5_1-231_: p = 0.023; NS5_673-903_: p <0.0001) and the median is shown. **(B)** Flow cytometric analysis of splenocytes restimulated with TBEV peptide pools. Representative FACS plots gated on live CD3^+^ CD4^+^ T cells show IFN-γ in LGTV infected mice in response to TBEV NS3_205-419_ and NS5_673-903_ peptide pool restimulation. *p<0.05, **p<0.01 and ****p<0.0001.

As for antibodies induced by LGTV infection, the LGTV infection induced T cell response and its cross-reactivity to TBEV antigens were analyzed. To this end, we restimulated splenocytes from control and LGTV infected mice with 15-mer synthetic peptide pools of C, E, NS1, NS3 and NS5 proteins of TBEV. These peptide pools were designed with 11 amino acid overlaps, hence ensuring that none of the CD4^+^ and CD8^+^ T cell epitopes were missed out. Frequencies of IFN-γ producing effector T cells were determined using IFN-γ ELISpot. A high frequency of T cells that specifically cross-reacted with epitopes within E, NS3 and NS5 proteins of TBEV was observed (**Figure 2A**). As indicated in **Figure 2A**, the most prominent response was observed upon restimulation with the peptide pool that encompasses the amino acid (aa) sequence 205-419 of NS3 and the C terminal region (aa673-903) of the NS5 protein. Furthermore, flow cytometric evaluation of splenocytes revealed that CD4^+^ T cells were the major source of IFN-γ producing cells more prominently in response to NS3_205-419_ and NS5_673-903_ restimulation (**Figure 2B**). Nevertheless, we could not detect CD8^+^ T cell effectors in response to the TBEV peptides restimulation.

### 3.3 Serum from LGTV protects mice against TBEV challenge

**Figure 3:**
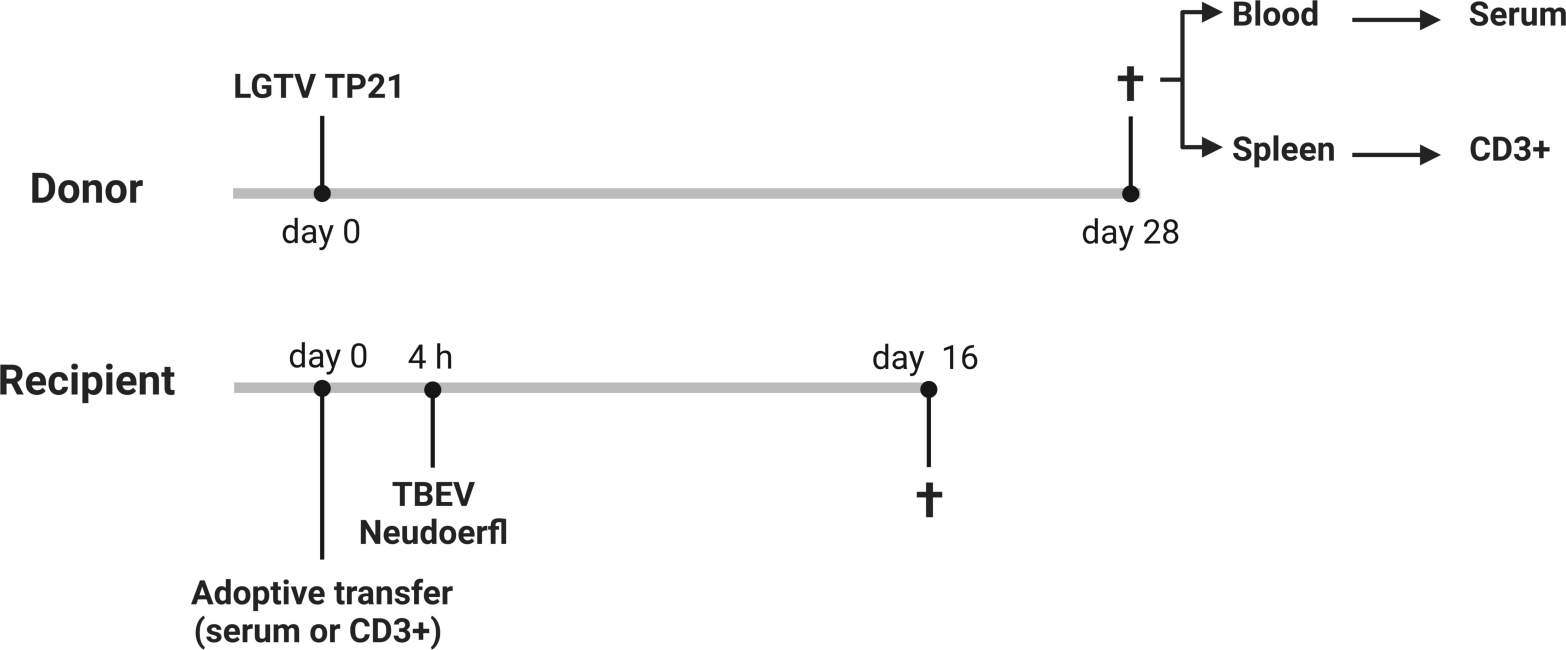
Schematic representation of adoptive transfer experiments. 6-8 weeks old control or LGTV infected donor mice (n=5) were sacrificed at 28 dpi and the sera and spleens were collected. 200 µl of pooled serum or 2.53x10^7^ CD3^+^ T cells from control or LGTV infected mice were administered i.p. into 10-12 weeks old naïve recipient mice (n=5). After 4 h, these mice were challenged with TBEV Neudoerfl (created with BioRender.com).

We demonstrated that LGTV infected mice developed VN antibodies that cross-react with TBEV. To determine if the cross-reactive antibodies confer protection to mice against lethal TBEV challenge, we adoptively transferred sera collected from control or LGTV infected mice into naïve BL6 mice prior to TBEV exposure (**Figure 3**; **Supplementary Figures S1A, B**).

**Figure 4:**
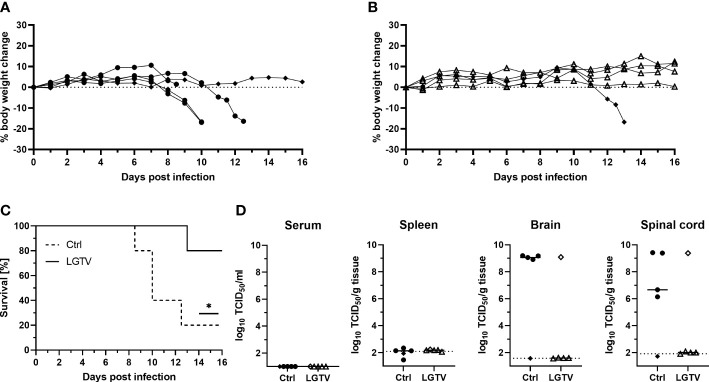
Immune serum from LGTV infected mice protects mice against subsequent TBEV challenge infection. Serum pooled from the respective group was i.p. injected into recipient mice (n=5). Following TBEV challenge infection, body weight of mice that received **(A)** control serum or **(B)** LGTV immune serum was monitored over a period of 16 dpi. Mice were taken out of experiment when the HEP was reached. **(C)** Survival curve plotted for the recipient mice that received control serum (dotted line) or serum from LGTV infected mice (solid line). Significant difference was analyzed by log rank test (p = 0.033). **(D)** TCID50 was performed on serum and tissue homogenates to determine the viral load in serum, spleen, brain and spinal cord of control (•) or LGTV (Δ) serum recipients collected at the day of sacrifice. Median values are shown. Mice that differed in their clinical state from other mice in the control and LGTV serum recipient group are highlighted as rhombus shaped symbols. *p<0.05.

Body weight loss and development of clinical signs were assessed daily and mice were sacrificed when the HEP was reached. Following TBEV challenge, the majority of mice (4 out of 5) that had received control sera continuously lost weight (**Figure 4A**) starting from 6 dpi and did not recover thereafter (**Figure 4C**). As the disease progressed, they displayed piloerection, decreased activity, kyphosis and signs of abdominal discomfort. These mice reached the HEP between 8-12 dpi and were subsequently sacrificed. Major HEP determinant in most cases was body weight loss reaching 20%. The one surviving mouse in this group did not display any clinical signs of disease and remained healthy until 16 dpi, which was the study endpoint. Contrarily, four out of five mice from the group that received sera from LGTV infected donors were completely protected and remained healthy without any visible clinical signs until 16 dpi (**Figures 4B, C**). Only one mouse in this group started losing weight at around 11 dpi and reached the HEP at 13 dpi with severe signs of disease characterized by extreme body weight loss, dulled fur with slightly hunched back, reduced activity and neurological signs manifested as spinal ataxia. Additionally, virus burden and distribution in the serum, spleen, spinal cord and brain was determined in these mice. No infectious virus was found in serum and spleen in any of these mice, suggesting absence of viremia at the time of sacrifice (**Figure 4D**). However, high TBEV infectivity titers were detected in the brain and spinal cord of all mice that reached the HEP including the one mouse that received serum from LGTV infected donors. TCID50 results confirm absence of infectious TBEV in brain and spinal cord of the remaining four healthy recipient mice from the LGTV serum group and one survivor from the control group (**Figure 4D**). Detection of viral genome copies by real time quantitative RT-PCR also yielded similar results with the exception of one survivor in the LGTV serum recipient group where infectious TBEV was not detected in TCID50 analysis but low levels of viral RNA (~10^5^ copies/gram tissue) could be found in the brain but not in the spinal cord (**Supplementary Figure S2**). Similarly, viral genome copies but not infectious virus could be detected in spleen and brain of the only survivor of the control serum recipient group. Overall, the results further confirm that LGTV antibodies cross-react with TBEV antigens and efficiently protect mice from lethal TBEV infection.

### 3.4 T cells from LGTV infected mice did not confer protection against TBEV challenge

**Figure 5:**
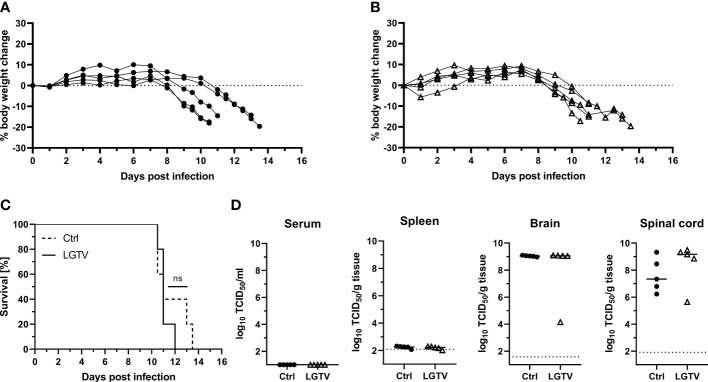
T cells from LGTV infected mice do not protect mice against subsequent TBEV challenge infection. CD3^+^ T cells purified from control or LGTV infected mice (n=5) were i.p. administered into naïve recipient mice (n=5). Following TBEV challenge infection, body weight of mice that received T cells of **(A)** control or **(B)** LGTV infected mice was monitored over a period of 16 dpi. Mice were taken out when they reached the HEP. **(C)** Survival curve plotted for the recipient mice that received control CD3^+^ T cells (dotted line) or CD3^+^ T cells from LGTV infected mice (solid line). Significant difference was analyzed by log rank test (p = 0.4389). **(D)** TCID50 was performed on serum and tissue homogenates to determine the viral load in serum, spleen, brain and spinal cord of control (•) or LGTV (Δ) CD3^+^ T cell recipients collected at the day of sacrifice. Median values are shown.

To further investigate the contribution of T cells induced by LGTV infection in protecting mice against TBEV infection, we adoptively transferred CD3^+^ T cells sorted from the spleens of either control or LGTV infected mice into naïve recipient mice prior to TBEV challenge (**Figure 3**; **Supplementary Figure S1C**). Purity of transferred T cells was determined by flow cytometry before transfer and was approximately 97% (**Supplementary Figure S3**). To ensure that the frequency of T cells actual reflected our previously published vaccination-challenge experiments, we transferred T cells isolated from five donor mice into five recipient mice. Unexpectedly, all mice that received T cells either from controls or from LGTV infected donors developed severe illness and succumbed to infection between 10-13 dpi (**Figure 5C**). Irrespective of the source of transferred T cells, the recipient mice displayed weight loss starting 7 dpi and never recovered thereafter (**Figures 5A, B**). Furthermore, the viral load in the brain and spinal cord between these two recipient groups was similar (**Figure 5D**, **Supplementary Figure S2B**) suggesting the inability of T cells to control the ongoing infection in the absence of serum antibodies.

### 3.5 Histopathological changes of the central nervous system

**Figure 6:**
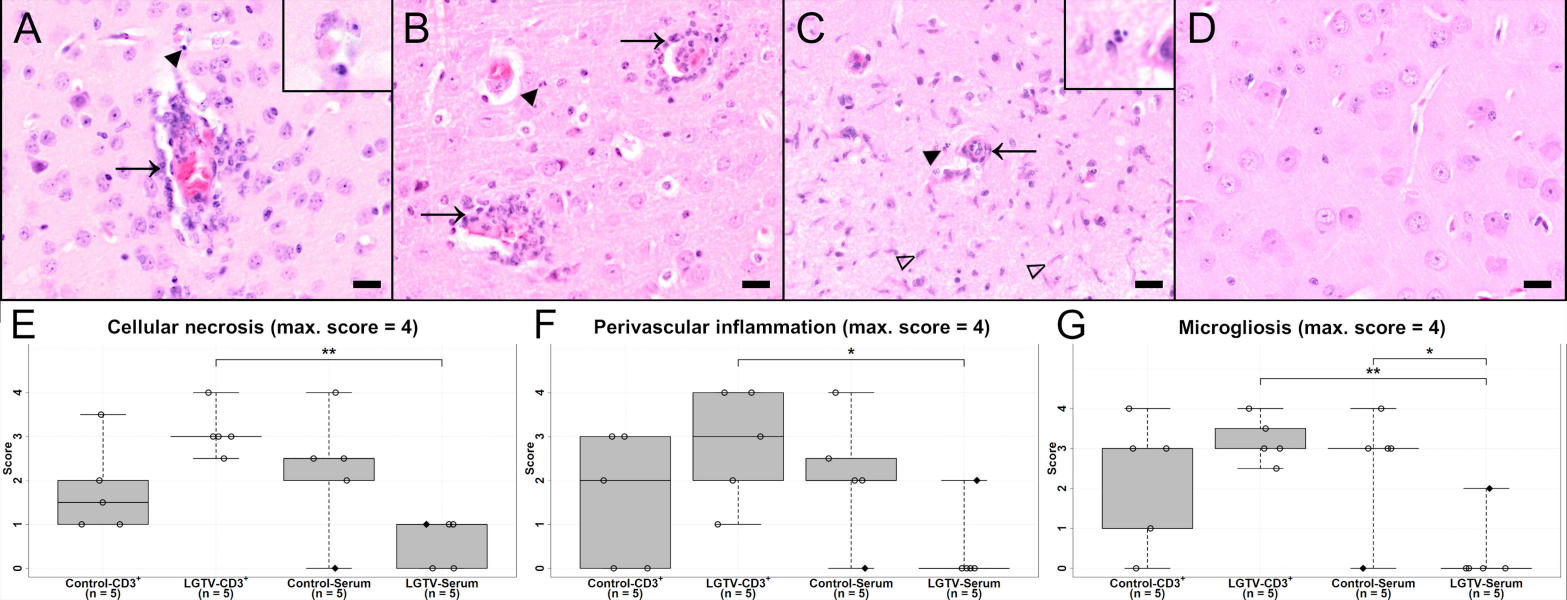
H&E stained sections of cerebral cortex of one representative mouse from each group **(A)** CD3^+^ T cell transfer from control donors; **(B)** CD3^+^ T cell transfer from LGTV infected donors; **(C)** serum transfer from control donors; **(D)** serum transfer from LGTV infected donors. In **(A–C)** signs of inflammation with infiltration of damaged blood vessels with inflammatory cells (arrow, **(A)**, **(B)**), perivascular inflammation as well as cell necrosis (arrowhead and inserts) and microgliosis (blank arrowhead, **(C)**) are visible. In the cerebral cortex of the mouse that had received serum from LGTV infected donors, no histopathological changes are observed **(D)**. Scale bars: 20 µm. **(E–G)** display box plots of the scoring values for “cellular necrosis” **(E)**, “perivascular inflammation” **(F)** and “microgliosis” **(G)** of cerebral cortex for each experimental group. Significant differences detected by pairwise Wilcoxon rank-sum tests after non-parametric ANOVA and multiple testing adjustment are indicated by asterisks (* p < 0.05; ** p< 0.01). Mice that differed in their clinical state from other mice in the control and LGTV serum recipient group are highlighted as rhombus shaped dots in the box plots.

The most striking histopathological changes observed in H&E stained brain sections of affected mice comprised cellular necrosis, microgliosis, perivascular inflammation and vasculitis in the brain parenchyma mostly confined to the grey matter and including the leptomeninx (**Figures 6A-G**). ANOVA for these parameters resulted in significant interaction effects, meaning the effect of LGTV treatment was inverse for CD3^+^ and serum transfer. After multiple testing adjustment, this interaction effect remained only significant for “H&E brain cerebral cortex cellular necrosis” (**Supplementary Table S6**). Affected brains displayed neuronal and glial necrosis characterized by shrunken and hypereosinophilic cells with karyorrhectic, karyolytic and pyknotic nuclei. Areas of necrosis were accompanied by an increased number of activated microglia/macrophages and T cells as well as neuronophagia. In addition, inflammatory infiltrates were predominantly found perivascularly and consisted mostly of lymphocytes, macrophages and scattered neutrophils. Additionally, a fibrinoid, non-leukocytoclastic vasculitis of small to medium sized blood vessels characterized by loss of vascular wall integrity with an infiltration of the destructed vascular wall with inflammatory cells was noticed.

All brain regions were scored according to the semiquantitative scoring scheme (**Supplementary Table S3**) and revealed similar findings. Therefore, three representative, consistently affected brain regions (olfactory bulb, cerebral cortex and hippocampus) were selected for more detailed analysis. Since statistical significances displayed high variations across brain regions and parameters, focus was set on these brain regions. Statistical scores as well as significant values for cerebral cortex and scoring categories including H&E and immunohistochemical evaluation are depicted in **Figure 6**; **Supplementary Figure S4**, **S5** and **Supplementary Table S6**.

In H&E stained sections, mice of the control-CD3^+^, LGTV-CD3^+^ and control-serum groups displayed more severe histopathological changes within the brain than mice that had received serum from LGTV infected donors (**Figure 6**; **Supplementary Figure S4**). Significant differences within the olfactory bulb for the scoring categories microgliosis, cellular necrosis and vascular inflammation were observed (**Supplementary Figures S4A, C**). The scores related to cellular necrosis differed significantly between the LGTV-CD3^+^ and the LGTV-serum group (**Supplementary Figure S4A**; p = 0.008) as well as the control-CD3^+^ and the LGTV-serum group (**Supplementary Figure S4A**; p = 0.018). A significant difference for microgliosis was detected between LGTV-CD3^+^ and LGTV-serum group (**Supplementary Figure S4C**; p = 0.008). Vascular inflammation scores differed significantly between control-serum and LGTV-serum group (p = 0.048). Within the cerebral cortex, mice from the LGTV-serum group displayed significantly lower scores for cellular necrosis of cerebral cortex than mice from the LGTV-CD3^+^ group (**Figure 6E**; p = 0.008). Furthermore, scores of the LGTV-serum group for microgliosis of cerebral cortex were significantly lower in comparison to LGTV-CD3^+^ (p = 0.008) and control-serum group (**Figure 6G**; p = 0.048). Perivascular inflammation of cerebral cortex scores were significantly different between LGTV-CD3^+^ group and LGTV-serum group (**Figure 6F**; p= 0.024). Overall, lowest scores were achieved by the group that had received serum from LGTV infected donors, although one mouse that presented clinical signs from this group displayed histopathological lesions in the brain (**Figures 6E-G**). In the hippocampus, a significant difference for microgliosis was observed between LGTV-CD3^+^ group and LGTV-serum group (**Supplementary Figure S4F**; p = 0.008).

**Figure 7:**
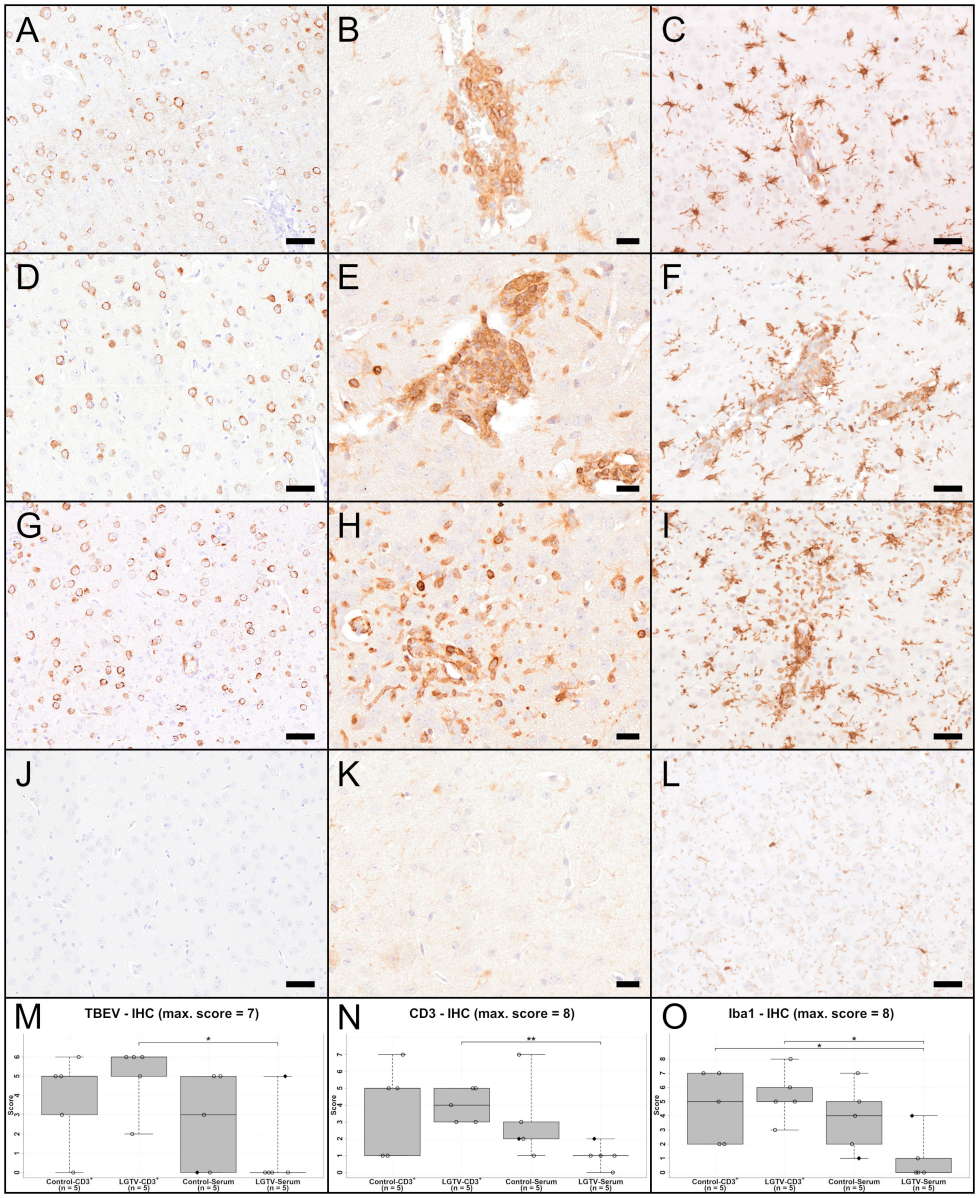
IHC of cerebral cortex for TBEV **(A, D, G, J)**, T cell marker CD3 **(B, E, H, K)** and microglia/macrophage marker Iba1 **(C, F, I, L)** of one representative mouse from each group **(A–C)**: CD3^+^ T cell transfer from control donors; **(D–F)**: CD3^+^ T cell transfer from LGTV infected donors; **(G–I)**: serum transfer from control donors; **(J–L)**: serum transfer from LGTV infected donors). TBEV E protein is detectable in the cytoplasm of neurons in all mice **(A, D, G)** except the mouse in the serum transfer group from LGTV infected donor mice **(J)**. CD3-positive T cells are detectable in the vascular wall as well as the perivascular space of affected mice **(B, E, H)**, while no immunoreaction is visible in the mouse of the serum transfer from LGTV infected donor mice **(K)**. There is distinct increase in staining intensity and number of Iba1-positive microglia/macrophages indicating activation and proliferation of microglia and/or proliferation/infiltration of macrophages in affected mice **(C, F, I)** in comparison to the mouse of the serum transfer group from LGTV infected donor mice in which microglia are normal-sized with fine processes **(L)**. Scale bar **(A, D, G, J, C, F, I, L)**: 50 µm; scale bar **(B, E, H, K)**: 20 µm. **(M–O)** display box plots of the scoring values of IHC for TBEV (TBEV-IHC; **(M)**, IHC for CD3 (Iba1-IHC; **(N)** and IHC for microglia/macrophages marker Iba1 (Iba1-IHC; **(O)** of cerebral cortex for each experimental group. Significant differences detected by pairwise Wilcoxon rank-sum tests after non-parametric ANOVA and multiple testing adjustment are indicated by asterisks (* p < 0.05; ** p< 0.01). Mice that differed in their clinical state from other mice in the control and LGTV serum recipient group are highlighted as rhombus shaped symbols in the box plots.

IHC using an antibody directed against the TBEV E protein **Figures 7A, D, G, J**) revealed the presence of high virus burden in the brain of all mice that succumbed to TBEV infection (15/20) irrespective of their treatment regimen (**Figures 7A, D, G**; **Supplementary Figures S5A, D**). TBEV antigen was mostly found in the cytoplasm of cells that appeared to be neurons according to distribution and morphology. This is consistent with previous findings (24). In four mice from the LGTV infected serum recipient group, no TBEV E protein was detected immunohistochemically. The only diseased mouse in this group where the histopathological changes were evident also showed positive immunoreactivity for TBEV E protein in the brain (**Figure 7M**). This is supported by statistical analysis. Scores of IHC-TBEV in the olfactory bulb differed significantly between the control-CD3^+^ and the LGTV-serum group (**Supplementary Figure S5A**; p = 0.018). Additionally, the cerebral cortex ANOVA yielded that IHC-TBEV scores were significantly higher in CD3^+^ T cell recipients than in serum recipients. Specifically, TBEV-IHC scores of the LGTV-serum group were significantly lower than scores of mice from LGTV-CD3^+^ group (**Figure 7M**; p = 0.024).

IHC for CD3 (**Figures 7B, E, H, K**) revealed that CD3^+^ T cells were infiltrating the perivascular space as well as the vascular wall of affected brains (**Figures 7B, E, H**). The olfactory bulb showed significant differences for CD3 immunoreactivity between control-CD3^+^ and LGTV-serum group (p = 0.036), between LGTV-CD3^+^ and LGTV-serum group (p = 0.008) and control-serum and LGTV-serum group (**Supplementary Figure S5B**; p = 0.024). Furthermore, IHC-CD3 scores in the cerebral cortex of the LGTV-serum group were significantly lower than scores of mice from LGTV-CD3^+^ group (**Figure 7N**; p = 0.008).

IHC for the microglia/macrophage marker Iba1 (**Figures 7C, F, I, J**) confirmed the presence of assumed microgliosis in brains with histopathological changes described above characterized by increased staining intensity and increased numbers of microglia/macrophages (**Figures 7C, F, I**, **Supplementary Figures S5C, F**). Statistical analysis revealed that IHC-Iba1 (vascular/perivascular + parenchymal) scores in the cerebral cortex of the LGTV-serum group were significantly lower than scores of mice from the LGTV-CD3^+^ group (**Figure 7O**; p = 0.016) and of mice from the control-CD3^+^ group (**Figure 7O**; p = 0.032).

### 3.6 Severe gastrointestinal pathology observed post TBEV challenge

**Figure 8:**
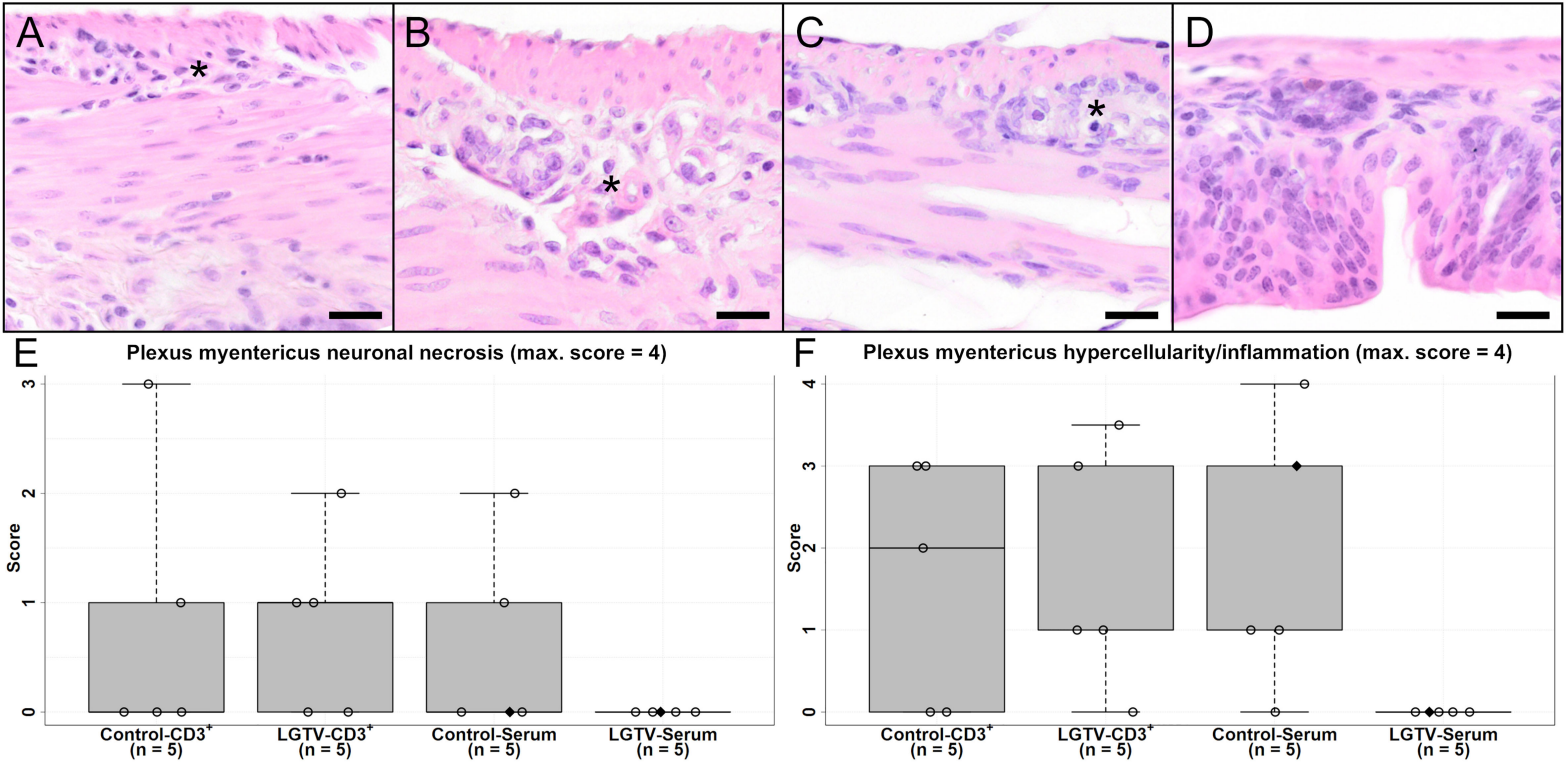
H&E stained sections of caecum of one representative mouse from each group **(A)** CD3^+^ T cell transfer from control donors; **(B)** CD3^+^ T cell transfer from LGTV infected donors; **(C)** serum transfer from control donors; **(D)** serum transfer from LGTV infected donors). The *plexus myentericus* displays varying degrees of ganglioneuritis in **(A–C)** (asterisk). No histopathological changes in the *plexus myentericus* of the mouse from the serum transfer of LGTV infected donor group **(D)** are detectable. Scale bars: 20 µm. **(E, F)** display box plots of the scoring values for *plexus myentericus* neuronal necrosis **(E)** and *plexus myentericus* hypercellularity/inflammation **(F)** of caecum for each group. Mice that differed in their clinical state from other mice in the control and LGTV serum recipient group are highlighted as rhombus shaped symbols in the box plots.

**Figure 9:**
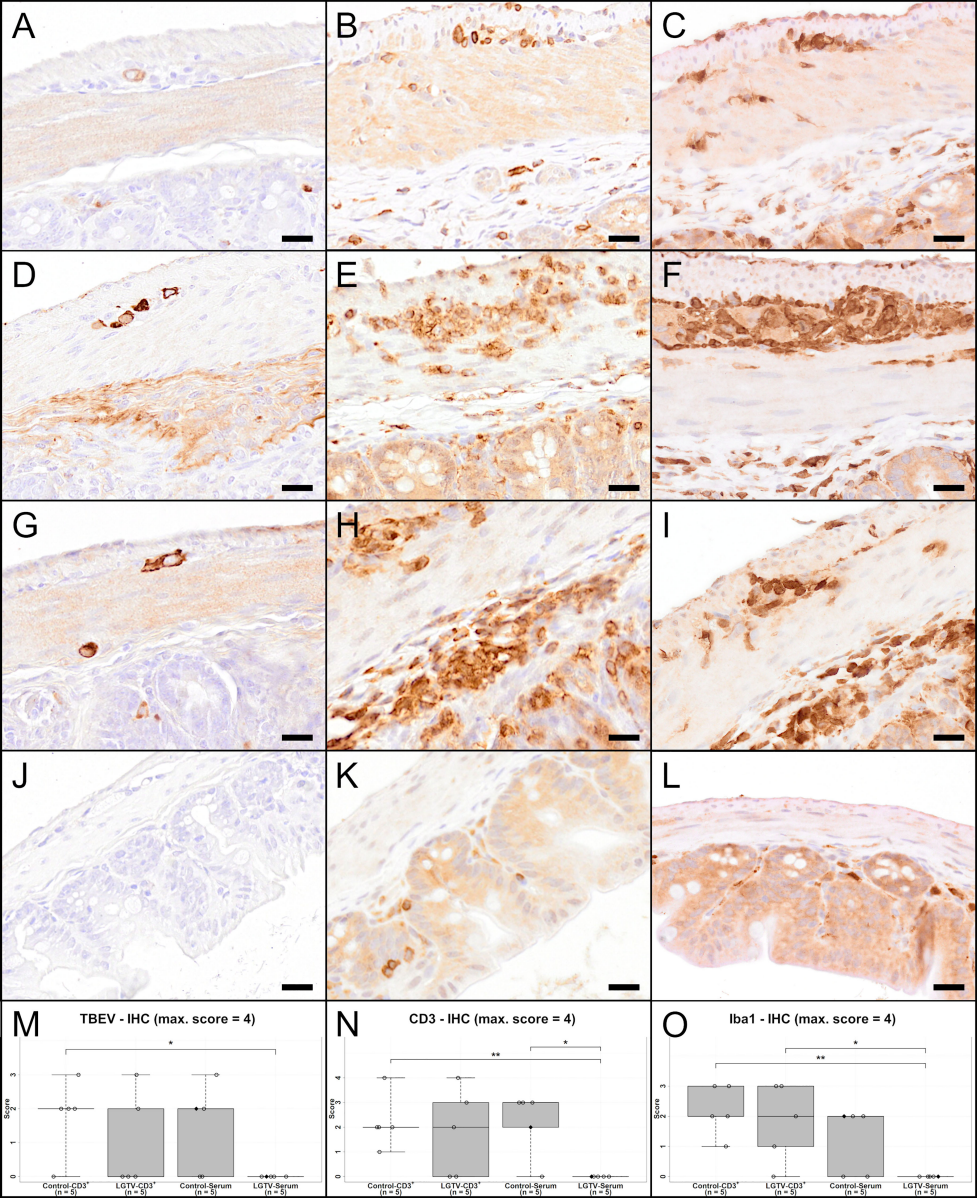
IHC of caecum for TBEV **(A, D, G, J)**, T cell marker CD3 **(B, E, H, K)** and microglia/macrophage marker Iba1 **(C, F, I, L)** of one representative mouse from each group **(A–C)** CD3^+^ T cell transfer from control donors; **(D–F)** CD3^+^ T cell transfer from LGTV infected donors; **(G–I)** serum transfer from control donors; **(J–L)** serum transfer from LGTV infected donors). TBEV E protein is detectable in the cytoplasm of neurons in the *plexus myentericus* and *submucosus* in all mice **(A, D, G)** except the ones in the group of the serum transfer from LGTV infected donor mice **(J)**. Ganglioneuritis in affected mice is characterized by CD3-positive T cell infiltration in both plexus **(B, E, H)**. No CD3-immunoreaction in the plexus of the serum transfer from LGTV infected donor mice is detectable **(K)**. In addition, an infiltration of plexus with Iba1-positive macrophages in affected mice **(C, F, I)** in comparison to mice of the group serum transfer from LGTV infected donor mice is detectable **(L)**. Scale bars: 20 µm. **(M–O)** display box plots of the scoring values of IHC for TBEV (IHC-TBEV; **(M)**, IHC for CD3 (IHC-CD3; **(N)** and IHC for macrophage marker Iba1 (Iba1-IHC; **(O)**. Significant differences detected by pairwise Wilcoxon rank-sum tests after non-parametric ANOVA and multiple testing adjustment are indicated by asterisks (* p < 0.05; ** p< 0.01). Mice that differed in their clinical state from other mice in the control and LGTV serum recipient group are highlighted as rhombus shaped symbols in the box plots.

Significant body weight loss (> 20%) following TBEV challenge was a prominent characteristic in all mice that reached the HEP. Analysis of H&E stained sections from duodenum, jejunum, ileum, caecum, colon and rectum revealed ganglioneuritis of the *myenteric* and *submucosal plexus* of varying degree in all groups except the LGTV-serum recipient group (**Figure 8**). Neurons in injured ganglia displayed signs of neuronal necrosis and furthermore, an infiltration with inflammatory cells and/or hyperplasia of resident immune cells was observed. After evaluation and scoring of all intestinal regions, ileum, caecum and colon displayed consistent alterations in affected mice and were therefore analyzed in more detail. Statistical results of the H&E as well as IHC scorings of the region caecum are shown in **Figures 8E, F**, **9M-O**; **Supplementary Figures S6**, **S7** and **Supplementary Table S6**. In detail, statistical significances of ileum for *plexus myentericus* hypercellularity/inflammation were observed between control-CD3^+^ and control-serum group (p = 0.048) and control-serum and LGTV-serum group (**Supplementary Figures S6A, B**; p = 0.008).

Accordingly, TBEV E protein was detected by IHC in the intestine of all groups (**Figures 9A, D, G**) except the LGTV-serum group (**Figure 9J**). Viral antigen was localized cytoplasmically in neurons of the *plexus myentericus* and *submucosus*. Specifically, IHC-TBEV scores in the caecum of the LGTV-serum group were significantly lower than scores of mice from the control-CD3^+^ group (**Figure 9M**; p = 0.048). IHC-TBEV scores of ileum and colon are displayed in **Supplementary Figures S7A, D**.

IHC for the T cell marker CD3 revealed an infiltration of affected ganglia with T cells in control-CD3^+^, LGTV-CD3^+^ and control-serum mice (**Figures 9B, E, H**) and lacking infiltration in LGTV-serum mice (**Figure 9K**). Significant differences for CD3-IHC of ileum were detected for *plexus myentericus* between control-CD3^+^ and LGTV-serum group (p = 0.024) as well as control-serum and LGTV-serum group (p = 0.008) and for *plexus submucosus* between control-serum and LGTV-serum group (**Supplementary Figure S7B**; p = 0.024). In the caecum (**Figure 9N**), significant differences for CD3-IHC scores in both *plexus submucosus* and in the *plexus myentericus* were observed between control-CD3^+^ and LGTV-serum group (p = 0.008) as well as control-serum and LGTV-serum group (**Supplementary Figure S7E**; p = 0.048).

Additionally, there was an infiltration/increased number of Iba1-positive cells (**Figures 9C, F, I, J**) with significant differences of Iba1-IHC scores in the *plexus myentericus* of ileum between control-CD3^+^ and LGTV-serum group (p = 0.008), LGTV-CD3^+^ and LGTV-serum group (p = 0.048) and control-serum and LGTV-serum group (**Supplementary Figure S7C**; p = 0.048). In the *plexus myentericus* of caecum, significant differences between control-CD3^+^ and LGTV-serum group (p = 0.008) as well as LGTV-CD3^+^ and LGTV-serum group (p = 0.048) were observed (**Figure 9O**). The Iba1-IHC scores of colon differed significantly in the *plexus myentericus* between control-serum and LGTV-serum group (p = 0.008) and in the *plexus submucosus* between LGTV-CD3^+^ and LGTV-serum group (**Supplementary Figure S7F**; p = 0.048).

The fact that mice displaying ganglioneuritis together with detection of TBEV E protein within neurons of ganglia and inflammatory cell infiltration with T cells could represent another factor leading to severe TBEV-associated disease in addition to CNS pathology.

## 4 Discussion

Using a mouse model of TBEV infection, we demonstrated that effectors of adaptive immunity induced upon LGTV infection are cross-reactive with TBEV. Nevertheless, only transfer of serum but not T cells from LGTV infected donor mice into naïve recipient mice protected against disease induced by subsequent TBEV challenge. Our study defines antibodies as a major correlate of protection induced by LGTV infection that protects against TBEV infection and prevents the mice from developing severe disease.

Although safety concerns have been associated with LGTV immunization in the past, as it is not fully apathogenic to humans, it still indicates potential for live-attenuated virus vaccines against TBE. Pre-existing immunity can either positively or negatively influence the outcome of subsequent infection with a heterologous flavivirus (1, 5, 7, 25, 26). Therefore, immune correlates induced by LGTV infection in providing either protective immunity or predisposing for more severe pathogenesis of TBEV infection needs to be further investigated.

A close antigenic relationship among flaviviruses allows antibodies induced against one virus to recognize and react with similar epitopes of another virus. Although, cross-reactivity is more pronounced among closely related members, there is evidence that this can also happen among distantly related flavivirus. For instance, a phylogenetic tree based on the amino acid sequence of the E protein shows that TBEV is closely related to other tick-borne virus such as Powassan virus and is distantly related to mosquito-borne ZIKV and DENV. Yet, antibodies induced against TBEV has been shown to cause slight enhancement of ZIKV infection *in vitro* (4). Similarly, serology of human cohorts vaccinated against TBEV and YFV show cross-neutralizing antibodies against Louping ill virus, WNV and DENV (27). Furthermore, it has been shown that immunity to YFV impairs the antibody response (5). Phillpotts and colleagues screened several mouse antibodies raised against closely related flaviviruses, including LGTV, and demonstrated antibody-dependent enhancement (ADE) of TBEV infectivity *in vitro* (28). Similarly, cross-reactive antibodies induced by ZIKV may also lead to enhancement of DENV infection (29). Since ADE to heterologous infections is not uncommon among flavivirus infections, we explored the effects of LGTV induced cross-reactive antibodies on TBEV infection.

It was encouraging to see that four among five mice that passively received immune serum from LGTV infected mice were completely protected and did not show any signs of disease or increased virus burden in the organs following lethal TBEV challenge. However, qPCR detected small amount of viral RNA in one of the four survivors from the LGTV serum recipient groups. This is in accordance with our previous work where we also found TBEV in the brain despite of protective immunity induced by prior LGTV infection (24). It is likely that virus that escapes immune detection in the periphery, enters and replicates within the CNS before the resident glial cells combined with infiltrating immune cells clear the infection. In contrast, viral RNA was detected in the spleen as well as in the brain of the only surviving mice in the control serum recipient group hinting at ineffective viral clearance from the periphery. Furthermore, histopathological examination did not show severe pathological lesions in the brain or gastrointestinal tract of surviving mice. Only one mouse of the LGTV serum group displayed histopathological lesions in the brain as well as TBEV-positive immunoreactivity. However, this mouse did not show significant pathological lesions within the gastrointestinal tract (**Figures 9E, F**) and no TBEV-immunopositive reaction was observed (**Figures 9M**). TBEV neutralizing antibody titers in sera collected from LGTV infected mice were significantly lower as compared to LGTV neutralization titers. However, this was sufficient to block infection and prevent mice from developing TBEV induced disease. Further screening of immune sera to determine the specificity of these antibodies revealed that a significantly high proportion of these antibodies is directed against the epitopes located in domain III of the TBEV E glycoprotein and the NS1 protein. This is in line with other studies showing that type specific anti-E and anti-NS1 antibodies contribute to protection in experimental models of TBEV and other flavivirus infections (30–33). Therefore, it is noteworthy that anti-E and anti-NS1 antibodies induced by LGTV infection may also be contributing to cross-protective immunity against TBEV. Currently, it is unclear if cross-reactive antibodies to other viral proteins such as C, prM, NS3 and NS4 are also induced by LGTV as our LIPS screening was not able to detect them in the immune sera (data not shown).

Like antibodies, T cell cross-reactivity for flaviviral antigens has also been reported (34). Nevertheless, there is evidence that the proportion of such cross-reactive T cells may be lower than what is known for antibodies (35). Interestingly, we also observed that T cells from LGTV infected mice recognized and responded to TBEV antigens. Hence, we speculated that this translated into protective immunity as shown for other flavivirus infections (8, 36). For instance, CD4^+^ and CD8^+^ T cells primed against DENV are capable of protecting mice against ZIKV infection (26, 37). Similar cross-reactive immunity is also seen in humans vaccinated against JEV and YFV, whose T cells responded to DENV antigens (38). Nevertheless, in our experiments adoptive transfer of T cells from LGTV infected donor mice failed to protect naïve recipient mice from subsequent challenge with TBEV. Upon further characterization by flow cytometry, we found that IFN-γ producing cells in response to NS3 and NS5 peptide pools restimulation were CD4^+^ T cells. We could not detect any effector CD8^+^ T cell response (IFN-γ^+^ and/or granzyme B^+^) to any given peptide pool restimulation. These observations are interesting and could partly explain the inefficiency of T cell transfer from LGTV infected mice to provide protection. Antigen-specific CD8+ T cells are among the key effectors of cell-mediated antiviral immunity as they recognize infected cells and prevent subsequent virus replication. Their role is especially significant for intracellular pathogens like viruses, which establish themselves in tissues and are less accessible to antibodies (39). In the T cell recipient mice, the absence of specific antibodies combined with the inability of LGTV infection to prime for TBEV cross-reactive CD8^+^ T cell responses may have been responsible for the failure to prevent TBEV spread into the CNS.

On the other hand, the effectors of CD4^+^ T cells activate antiviral mechanisms by other cells and may play a significant role in viral clearance. Nevertheless, in CNS with less immune activity, increased presence of T cells can also be detrimental and contribute to neural tissue damage. Although we did not observe differences in the onset and clinical progression of disease between recipient mice that received T cells from control or LGTV infected donors, histopathological observations indicate pronounced microgliosis and cellular necrosis in all affected mice. Furthermore, presence of T cells in the vascular and perivascular regions possibly hint at their contribution in pathological neuroinflammatory processes in the CNS.

Another previously described key feature of this TBEV infection model is gastrointestinal pathology in majority of affected mice (40). This is most likely a consequence of ganglioneuritis in the *plexus myentericus* and *submucosus* leading to dysfunctionality of the gastrointestinal tract. Although histopathological evaluations show increased presence of T cells in these areas, it is rather inconclusive whether T cells contribute to neuronal necrosis. It must be noted that mice start showing weight loss around 6 dpi which also gives time to prime endogenous T cell repertoire. This may be the reason why no significant differences were observed between mice recipient of control serum, control CD3^+^ T cells and LGTV CD3^+^ T cells.

Based on the information obtained from this and a previous study (24), we conclude that LGTV-specific antibodies that recognize TBEV antigens are main contributors of protection. On the other hand, T cells in the absence of antibody-mediated virus control may contribute to pathological changes. This could be vital information for the future design of live vaccines against TBEV and other closely related flavivirus infections.

## Data availability statement

The raw data supporting the conclusions of this article will be made available by the authors, without undue reservation.

## Ethics statement

The animal study was reviewed and approved by Lower Saxony State Office for Consumer Protection and Food Safety.

## Author contributions

Conceptualization: CKP, AO, and GR. Methodology: JB, MKu, GS, MP-G, CKP, IZ, and CP. Formal analysis: JB, MKu, CKP, IZ, CP, WB, and MKi. Investigation: JB, MKu, CKP, IZ, CP, WB, and MKi. Resources: IS, IZ, CP, and WB. Writing—original draft preparation: JB, MKu, CKP, and IZ. Writing—review and editing: IZ, GS, MKi, MP-G, IS, CP, KJ, WB, GR, AO. Visualization: JB, MKu, CKP, and IZ. Supervision: CKP, AO, GR. Funding acquisition: AO, GR, and WB. All authors contributed to the article and approved the submitted version.

## Supplementary material

Supplementary Figure 1 TBEV cross-reactive antibodies and T cells of adoptively transferred serum and T cells. Pooled sera from control or LGTV infected donor mice (n=5) were tested for their ability to block the infection of **(A)** LGTV and **(B)** TBEV in VeroE6 and A549 cells, respectively. The graphs show the titer at which 100% virus neutralization is achieved (VNT100). **(C)** Splenocytes obtained from individual control (•) or LGTV (Δ) infected donor mice (n=5) were restimulated with TBEV-specific peptide pools and the frequency of IFN-γ producing cells was determined using ELISpot assay. The median is shown.

Supplementary Figure 2 Viral load in the organs of serum and T cell recipient mice that were challenged with TBEV. Real time quantitative RT-PCR was performed on total RNA isolated from serum and tissue homogenates collected at day of sacrifice of recipient mice which either received **(A)** serum or **(B)** CD3^+^ T cells from control (•) or LGTV (Δ) infected donor mice and were subsequently challenged with TBEV. The median is shown. Mice that differed in their clinical state from other mice in the control and LGTV serum recipient group are highlighted as rhombus shaped symbols

Supplementary Figure 3 Purity of adoptively transferred CD3^+^ T cell pools. Flow cytometric analysis of purified CD3^+^ T cells from control (upper panel) and LGTV (lower panel) donor groups prior to adoptive transfer to recipient mice. FACS plots are gated on live CD3^+^ T cells.

Supplementary Figure 4 Box plots of the hematoxylin and eosin (H&E) scoring values for “cellular necrosis” **(A, D)**, “perivascular inflammation” **(B, E)** and “microgliosis” **(C, F)** of olfactory bulb **(A-C)** and hippocampus **(D-F)** for each experimental group. Significant differences detected by pairwise Wilcoxon rank-sum tests after non-parametric ANOVA are indicated by asterisks (* p < 0.05; ** p< 0.01; *** p < 0.001). Mice that differed in their clinical state from other mice in the control and LGTV serum recipient group are highlighted as rhombus shaped symbols in the box plots.

Supplementary Figure 5 Box plots of the scoring values of immunohistochemistry for TBEV **(A, D)**, T cell marker CD3 **(B, E)** and microglia/macrophage marker Iba1 **(C, F)** of olfactory bulb **(A-C)** and hippocampus **(D-F)** for each experimental group. Significant differences detected by pairwise Wilcoxon rank-sum tests after non-parametric ANOVA are indicated by asterisks (* p < 0.05; ** p< 0.01; *** p < 0.001). Mice that differed in their clinical state from other mice in the control and LGTV serum recipient group are highlighted as rhombus shaped symbols in the box plots.

Supplementary Figure 6 Box plots of the hematoxylin and eosin (H&E) scoring values for “plexus myentericus neuronal necrosis” **(A, C)** and “plexus myentericus hypercellularity” **(B, D)** of ileum **(A, B)** and colon **(C, D)** for each experimental group. Significant differences detected by pairwise Wilcoxon rank-sum tests after non-parametric ANOVA are indicated by asterisks (* p < 0.05; ** p< 0.01; *** p < 0.001). Mice that differed in their clinical state from other mice in the control and LGTV serum recipient group are highlighted as rhombus shaped symbols in the box plots.

Supplementary Figure 7 Box plots of the scoring values of immunohistochemistry for TBEV **(A, D)**, T cell marker CD3 **(B, E)** and microglia/macrophage marker Iba1 **(C, F)** of ileum **(A-C)** and colon **(D-F)** for each experimental group. Significant differences detected by pairwise Wilcoxon rank-sum tests after non-parametric ANOVA are indicated by asterisks (* p < 0.05; ** p< 0.01; *** p < 0.001). Mice that differed in their clinical state from other mice in the control and LGTV serum recipient group are highlighted as rhombus shaped symbols in the box plots.
