# Supplementary material for: Cross-reactive antibodies against Langat virus protect mice from lethal tick-borne encephalitis virus infection
Source: Front Immunol. 2023 Feb 28;14:1134371. doi: 10.3389/fimmu.2023.1134371 (PMC10011100; doi:10.3389/fimmu.2023.1134371)
Supplement: Supplementary file 1 [file DataSheet_1.docx]

Supplementary Material

Cross-reactive antibodies against Langat virus protect mice from lethal tick-borne encephalitis virus infection

Mareike Kubinski^†^, Jana Beicht^†^, Isabel Zdora^†^, Giulietta Saletti, Magdalena Kircher, Monique Petry-Gusmag, Imke Steffen, Christina Puff, Klaus Jung, Wolfgang Baumgärtner, Guus F. Rimmelzwaan, Albert Osterhaus^#^, Chittappen Kandiyil Prajeeth^#*^

*** Correspondence:** Dr. Chittappen Kandiyil Prajeeth: prajeeth.chittappen.kandiyil@tiho-hannover.de

# Supplementary Data

Supplementary Table S1: **Clinical scoring of mice**

| **Category** | **Score** | **Criteria** | |
| --- | --- | --- | --- |
| Outer appearance | 0-3 | Appearance, fur | |
| Activity | 0-3 | Attention, curiosity, spontaneous and induced movements | |
| Movement | 0-4 | Movement process | |
| Body weight | 0-3 | Percentage of body weight loss | |
|  | 0-13 | Overall score | |
|  | | | |
| **Action** | | | **Score** |
| Monitoring twice a day | | | 3-7 |
| Euthanasia (humane endpoint) | | | 8, maximal score in one category |

Supplementary Table S2: **Peptide pools of TBEV C, E, NS1, NS3 and NS5 proteins**

| **Protein** | **Pool** | **Total number of peptides** |
| --- | --- | --- |
| C | C_1-117_ | 27 |
| E | E_1-255_ | 61 |
|  | E_245-496_ | 60 |
| NS1 | NS1_1-183_ | 43 |
|  | NS1_173-352_ | 42 |
| NS3 | NS3_1-215_ | 51 |
|  | NS3_205-419_ | 50 |
|  | NS3_409-621_ | 50 |
| NS5 | NS5_1-231_ | 55 |
|  | NS5_221-459_ | 55 |
|  | NS5_449-683_ | 55 |
|  | NS5_673-903_ | 54 |

Supplementary Table S3: **Scoring of H&E stained brain sections**

| **S.nr** | **Description** | **Observation** | **Score** |
| --- | --- | --- | --- |
| 1 | Meninges | no inflammation | 0 |
|  |  | “minimal” with single inflammatory cells | 1 |
|  |  | “mild” with 1 layer of perivascular inflammatory cells | 2 |
|  |  | “moderate” with 2-3 layers of perivascular inflammatory cells | 3 |
|  |  | severe” with >3 layers of perivascular inflammatory cells | 4 |
| 2 | Perivascular inflammation | no inflammation | 0 |
|  |  | “minimal” with single inflammatory cells | 1 |
|  |  | “mild” with 1 layer of perivascular inflammatory cells | 2 |
|  |  | “moderate” with 2-3 layers of perivascular inflammatory cells | 3 |
|  |  | severe” with >3 layers of perivascular inflammatory cells | 4 |
| 3 | Vascular inflammation with inflammatory cells infiltrating the vascular wall | no inflammation | 0 |
|  |  | “mild” with single inflammatory cells (1-5 cells within the vascular wall) | 1 |
|  |  | “moderate” infiltration with inflammatory cells (6-10 cells within the vascular wall) | 2 |
|  |  | “severe” infiltration with inflammatory cells almost obscuring the vascular wall completely | 3 |
| 4 | Vascular lesions (perivascular edema, hemorrhage and fibrinoid necrosis) | not present | 0 |
|  |  | present | 1 |
| 5 | Microgliosis (hyperplasia and/or hypertrophy of microglia/macrophages) | no microgliosis | 0 |
|  |  | “minimal” with single activated microglia/macrophages | 1 |
|  |  | “mild” multifocal microgliosis (1 layer of perivascular located activated microglia/macrophages) | 2 |
|  |  | “moderate” multifocal microgliosis (2-3 layers of perivascular located activated microglia/macrophages) | 3 |
|  |  | “severe” multifocal microgliosis (> 3 layers of perivascular located activated microglia/macrophages) | 4 |
| 6 | Cellular necrosis (karyorrhexis, karyolysis, pyknosis and triangular shaped, hyperosinophilic, shrunken neurons) | no necrosis | 0 |
|  |  | “minimal” with single necrotic cells (1-5 necrotic cells per high power field (HPF)) | 1 |
|  |  | “mild” multifocal cellular necrosis with (6-10 necrotic cells per HPF) | 2 |
|  |  | “moderate” multifocal cellular necrosis (11-15 necrotic cells per HPF) | 3 |
|  |  | “severe” multifocal cellular necrosis (>15 necrotic cells per HPF) | 4 |

Supplementary Table S4: **Scoring of H&E stained intestinal sections**

| **S.nr** | **Description** | **Observation** | **Score** |
| --- | --- | --- | --- |
| 1 | Hypercellularity/inflammatory infiltrates within the *lamina propria* of the *tunica mucosa* | no inflammation | 0 |
|  |  | “minimal” with single inflammatory cells/hypercellularity (3-5 cells per HPF) | 1 |
|  |  | “mild” infiltration with inflammatory cells/hypercellularity (6-10 cells per HPF) | 2 |
|  |  | “moderate” infiltration with inflammatory cells/hypercellularity (11-20 cells per HPF) | 3 |
|  |  | “severe” infiltration with inflammatory cells/hypercellularity (>20 cells per HPF) | 4 |
| 2 | Necrosis of ganglion neurons in the *plexus submucosus* and *plexus myentericus* ganglia (karyorrhexis, karyolysis, pyknosis, hyperosinophilia, shrinkage of neurons). *Plexus submucosus* and *plexus myentericus* ganglia were scored separately. | no necrosis | 0 |
|  |  | “minimal” with single necrotic neurons (1 necrotic cell per HPF) | 1 |
|  |  | “mild” multifocal neuronal necrosis (2-3 necrotic cells per HPF) | 2 |
|  |  | “moderate” multifocal neuronal necrosis (4-5 necrotic cells per HPF) | 3 |
|  |  | “severe” multifocal neuronal necrosis (>5 necrotic cells per HPF) | 4 |
| 3 | Inflammatory infiltrates/hypercellularity in the *plexus submucosus* and *plexus myentericus* ganglia. *Plexus submucosus* and *plexus myentericus* ganglia were scored separately. | no inflammatory cells | 0 |
|  |  | “minimal” with single inflammatory cells (1-3 inflammatory cells per HPF) | 1 |
|  |  | "mild" infiltration with inflammatory cells/hypercellularity (4-7 inflammatory cells per HPF) | 2 |
|  |  | "moderate" infiltration with inflammatory cells/hypercelluarity (8-10 inflammatory cells per HPF) | 3 |
|  |  | "severe" infiltration with inflammatory cells/hypercelluarity (>10 inflammatory cells per HPF) | 4 |

Supplementary Table S5: **Scoring of immunohistochemical evaluation of sections**

| **S.nr** | **Detection of** | **Description** | **Observation** | **Score*** |
| --- | --- | --- | --- | --- |
| 1 | Virus in brain | A) TBEV E antigen signal | no immunopositive signal detectable | 0 |
|  |  |  | single cells immunopositive for TBEV (1-5 cells per HPF) | 1 |
|  |  |  | low numbers of cells immunopositive for TBEV (6-10 cells per HPF) | 2 |
|  |  |  | moderate numbers of cells immunopositive for TBEV (11-15 cells per HPF) | 3 |
|  |  |  | high numbers of cells immunopositive for TBEV (>15 cells per HPF) | 4 |
|  |  | B) Distribution of TBEV signal | focal | 1 |
|  |  |  | multifocal | 2 |
|  |  |  | diffuse | 3 |
| 2 | Virus in intestine (*plexus myentericus* and *submucosus*) | A) TBEV E antigen signal | no immunopositive signal detectable | 0 |
|  |  |  | single cells immunopositive for TBEV (1-5 cells per HPF) | 1 |
|  |  |  | low numbers of cells immunopositive for TBEV (6-10 cells per HPF) | 2 |
|  |  |  | moderate numbers of cells immunopositive for TBEV (11-15 cells per HPF) | 3 |
|  |  |  | high numbers of cells immunopositive for TBEV (>15 cells per HPF) | 4 |
| 3 | T cells in brain | A) CD3-positive signal (vascular/ perivascular appearance) | no CD3-positive cells | 0 |
|  |  |  | single CD3-positive cells | 1 |
|  |  |  | 1-3 layers of CD3-positive cells | 2 |
|  |  |  | 4-8 layers of CD3-positive cells | 3 |
|  |  |  | >8 layers of CD3-positive cells | 4 |
|  |  | B) Parenchymal | no CD3-positive cells | 0 |
|  |  |  | 1-3 CD3-positive cells per HPF | 1 |
|  |  |  | 4-7 CD3-positive cells per HPF | 2 |
|  |  |  | 8-10 CD3-positive cells per HPF | 3 |
|  |  |  | >10 CD3-positive cells per HPF | 4 |
| 4 | T cells in intestine | CD3-positive signal (*plexus myentericus* and *plexus submucosus*) | no CD3-positive cells | 0 |
|  |  |  | 1-3 CD3-positive cells per HPF | 1 |
|  |  |  | 4-7 CD3-positive cells per HPF | 2 |
|  |  |  | 8-10 CD3-positive cells per HPF | 3 |
|  |  |  | >10 CD3-positive cells per HPF | 4 |
| 5 | Microglia/ macrophages in brain | A) Iba1-positive cells | no increase in staining/hypertrophy of microglia/macrophages above the control background | 0 |
|  |  |  | single Iba1-positive cells/minimal increase in staining of Iba1-positive cells above the control background | 1 |
|  |  |  | 1-3 layers of perivascular Iba1-positive cells/mild increase in staining of Iba1-positive cells | 2 |
|  |  |  | 4-8 layers of perivascular Iba1-positive cells/moderate increase in staining of Iba1-positive cells | 3 |
|  |  |  | >8 layers of perivascular Iba1-positive cells/severe increase in staining of Iba1-positive cells | 4 |
|  |  | B) Distribution of Iba1-positive cells | focal | 1 |
|  |  |  | multifocal | 2 |
|  |  |  | diffuse | 3 |
| 6 | Macrophages in intestine | Iba1-positive cells | no Iba1-positive cells above control background | 0 |
|  |  |  | single Iba1-positive cells (3-5 cells per HPF) above control background | 1 |
|  |  |  | low number of Iba1-positive cells (6-10 cells per HPF) above control background | 2 |
|  |  |  | moderate number of Iba1-positive cells (11-20 cells per HPF) above control background | 3 |
|  |  |  | high number of Iba1-positive cells (>20 cells per HPF) above control background | 4 |
| * For those categories listed A and B the final score was obtained by adding A+B | | | | |
