# Supplementary material for: Cross-reactive antibodies against Langat virus protect mice from lethal tick-borne encephalitis virus infection
Source: Front Immunol. 2023 Feb 28;14:1134371. doi: 10.3389/fimmu.2023.1134371 (PMC10011100; doi:10.3389/fimmu.2023.1134371)
Supplement: Supplementary file 2 [file DataSheet_2.docx]

Supplementary Table S6: **Raw and for multiple testing adjusted *p*-values for transfer type (Serum or T-cells) and immunization (Control or LGTV) effect as well as for the interaction effect for selected parameters.**

| **Score** | **Raw *p*-values** | | | **Adjusted *p*-values** | | |
| --- | --- | --- | --- | --- | --- | --- |
|  | **Transfer type** | **Immunization** | **Transfer type x Immunization** | **Transfer type** | **Immunization** | **Transfer type x Immunization** |
| H&E Cerebral cortex cellular necrosis | **0.0196** | 0.7987 | **0.0031** | 0.1176 | 1.0000 | **0.0186** |
| H&E Cerebral cortex perivascular inflammation | 0.1086 | 0.6607 | **0.0281** | 0.4344 | 1.0000 | 0.0875 |
| H&E Cerebral cortex microgliosis | 0.0549 | 0.2648 | **0.0175** | 0.2745 | 1.0000 | 0.0875 |
| IHC TBEV Cerebral Cortex | **0.0105** | 0.8903 | 0.1429 | **0.0105** | 0.8903 | 0.1429 |
| IHC CD3 Cerebral Cortex | **0.0233** | 0.3525 | 0.0869 | **0.0233** | 0.3525 | 0.0869 |
| IHC Iba1 Cerebral Cortex vascular / perivascular + parenchymal | **0.0105** | 0.2708 | 0.0691 | **0.0105** | 0.2708 | 0.1382 |
| H&E Olfactory bulb cellular necrosis | **0.0003** | 0.3824 | 0.0556 | **0.0018** | 1.0000 | 0.2224 |
| H&E Olfactory bulb perivascular inflammation | 0.5605 | 0.3291 | 0.2275 | 1.0000 | 1.0000 | 0.4550 |
| H&E Olfactory bulb microgliosis | **0.0022** | 0.9681 | **0.0176** | **0.0110** | 1.0000 | 0.0880 |
| IHC TBEV Olfactory bulb | **0.0292** | 0.1294 | 0.8775 | 0.0292 | 0.1294 | 0.8775 |
| IHC CD3 Olfactory bulb | **0.0049** | 0.1096 | **0.0022** | **0.0049** | 0.1096 | **0.0022** |
| IHC Iba1 Olfactory bulb vascular / perivascular + parenchymal | 0.0620 | 0.0751 | 0.2291 | 0.1240 | 0.1502 | 0.2291 |
| H&E Hippocampus cellular necrosis | 0.0590 | 0.9307 | 0.0590 | 0.3540 | 1.0000 | 0.2650 |
| H&E Hippocampus perivascular inflammation | 0.0683 | 0.9659 | 0.1374 | 0.3540 | 1.0000 | 0.4122 |
| H&E Hippocampus microgliosis | 0.0607 | 0.8215 | **0.0391** | 0.3540 | 1.0000 | 0.2346 |
| IHC TBEV Hippocampus | 0.1174 | 0.8071 | 0.1174 | 0.1174 | 0.8071 | 0.1174 |
| IHC CD3 Hippocampus | 0.1130 | 0.6509 | 0.1617 | 0.1130 | 0.6509 | 0.1617 |
| IHC Iba1 Hippocampus vascular / perivascular + parenchymal | 0.0548 | 0.1688 | 0.0768 | 0.0548 | 0.3376 | 0.1046 |
| H&E Caecum plexus myentericus neuronal necrosis | 0.1918 | 0.5630 | 0.2377 | 0.8885 | 1.0000 | 0.9508 |
| H&E Caecum plexus myentericus hypercellularity/inflammation | 0.1777 | 0.1528 | 0.0527 | 0.8885 | 0.7430 | 0.2635 |
| IHC TBEV Caecum | 0.1830 | **0.0445** | 0.5548 | 0.1830 | **0.0445** | 0.5548 |
| IHC CD3 Caecum plexus myentericus | 0.1384 | **0.0283** | 0.1082 | 0.1384 | **0.0374** | 0.2164 |
| IHC CD3 Caecum plexus submucosus | 0.0488 | **0.0187** | 0.3022 | 0.0976 | **0.0374** | 0.3022 |
| IHC Iba1 Caecum plexus myentericus | **0.0036** | 0.0821 | 0.3674 | **0.0072** | 0.1642 | 0.7348 |
| IHC Iba1 Caecum plexus submucosus | 0.1999 | 0.6993 | 0.5896 | 0.1999 | 0.6993 | 0.5896 |
| H&E Ileum plexus myentericus neuronal necrosis | 0.4288 | 0.4288 | **0.0105** | 1.0000 | 1.0000 | **0.0420** |
| H&E Ileum plexus myentericus hypercellularity/inflammation | 0.8338 | 0.0660 | **0.0025** | 1.0000 | 0.3300 | **0.0125** |
| IHC TBEV Ileum | 0.1284 | 0.1284 | 0.6003 | 0.1284 | 0.1284 | 0.6003 |
| IHC CD3 Ileum plexus myentericus | 0.4438 | **0.0063** | **0.0109** | 0.8876 | **0.0126** | **0.0109** |
| IHC CD3 Ileum plexus submucosus | 0.8359 | **0.0465** | **0.0035** | 0.8876 | **0.0465** | **0.0070** |
| IHC Iba1 Ileum plexus myentericus | 0.1053 | **0.0422** | **0.0260** | 0.2106 | 0.0844 | 0.0520 |
| IHC Iba1 Ileum plexus submucosus | 0.1875 | 0.1045 | 0.5010 | 0.2106 | 0.1045 | 0.5010 |
| H&E Colon plexus myentericus neuronal necrosis | 0.9171 | 0.3822 | 0.2834 | 1.0000 | 0.9966 | 0.5668 |
| H&E Colon plexus myentericus hypercellularity/inflammation | 0.5667 | 0.1559 | 0.0984 | 1.0000 | 0.6236 | 0.2952 |
| IHC TBEV Colon | 0.6185 | 0.0542 | 0.2843 | 0.6185 | 0.0542 | 0.2843 |
| IHC CD3 Colon plexus myentericus | 0.1044 | **0.0150** | 0.2150 | 0.2088 | **0.0300** | 0.4300 |
| IHC CD3 Colon plexus submucosus | 0.4439 | 0.0591 | 0.3933 | 0.4439 | 0.0591 | 0.4300 |
| IHC Iba1 Colon plexus myentericus | 0.3397 | **0.0426** | 0.0666 | 0.3397 | 0.0852 | 0.1332 |
| IHC Iba1 Colon plexus submucosus | **0.0010** | **0.0020** | 0.5000 | **0.0116** | 0.6868 | 0.6147 |
