# Supplementary figures and images for: Cross-reactive antibodies against Langat virus protect mice from lethal tick-borne encephalitis virus infection
Source: Front Immunol. 2023 Feb 28;14:1134371. doi: 10.3389/fimmu.2023.1134371 (PMC10011100; doi:10.3389/fimmu.2023.1134371)

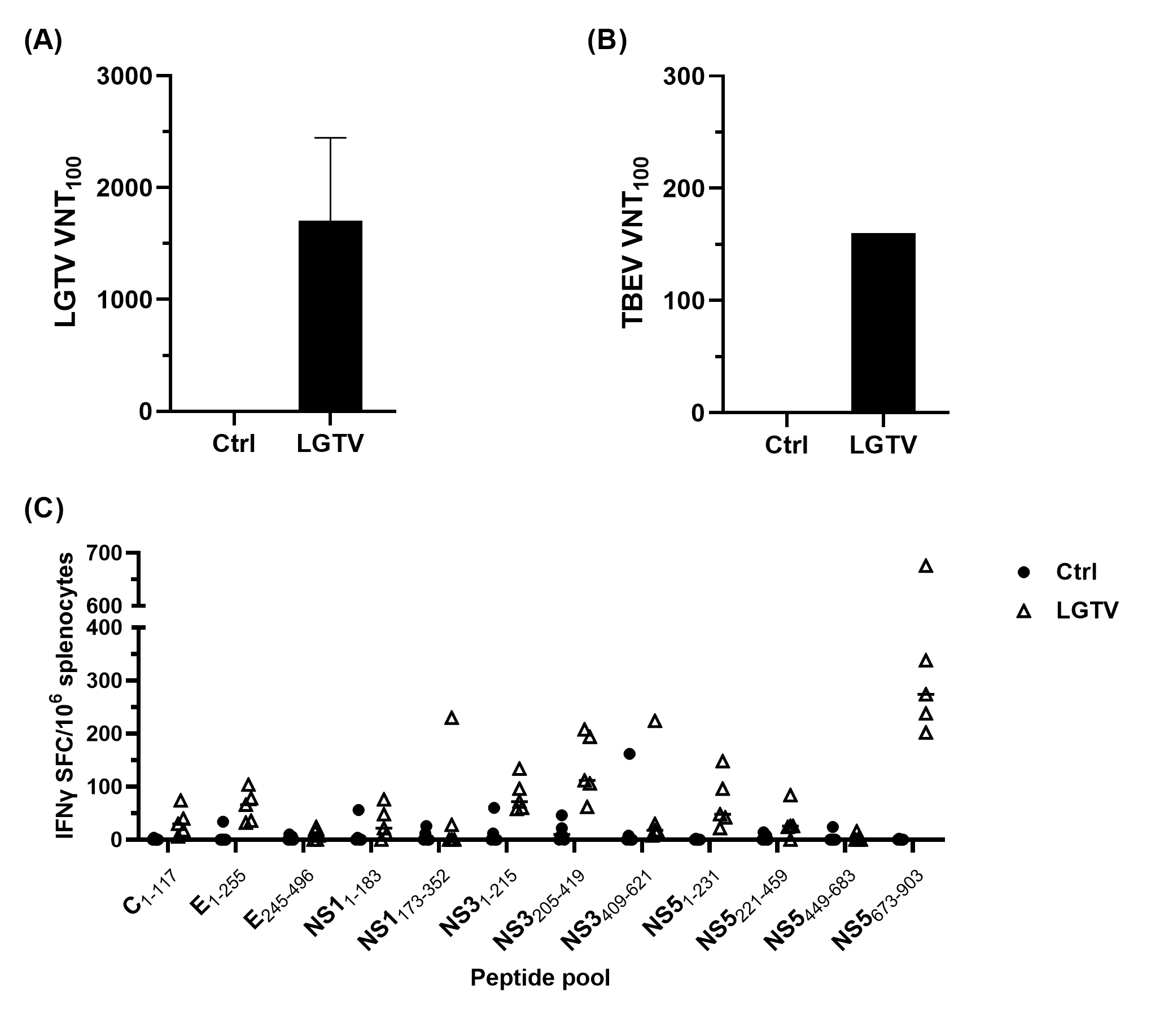

Supplement: Supplementary Figure 1 — TBEV cross-reactive antibodies and T cells of adoptively transferred serum and T cells. Pooled sera from control or LGTV infected donor mice (n=5) were tested for their ability to block the infection of (A) LGTV and (B) TBEV in VeroE6 and A549 cells, respectively. The graphs show the titer at which 100% virus neutralization is achieved (VNT100). (C) Splenocytes obtained from individual control (•) or LGTV (Δ) infected donor mice (n=5) were restimulated with TBEV-specific peptide pools and the frequency of IFN-γ producing cells was determined using ELISpot assay. The median is shown. [file Image_1.tif]

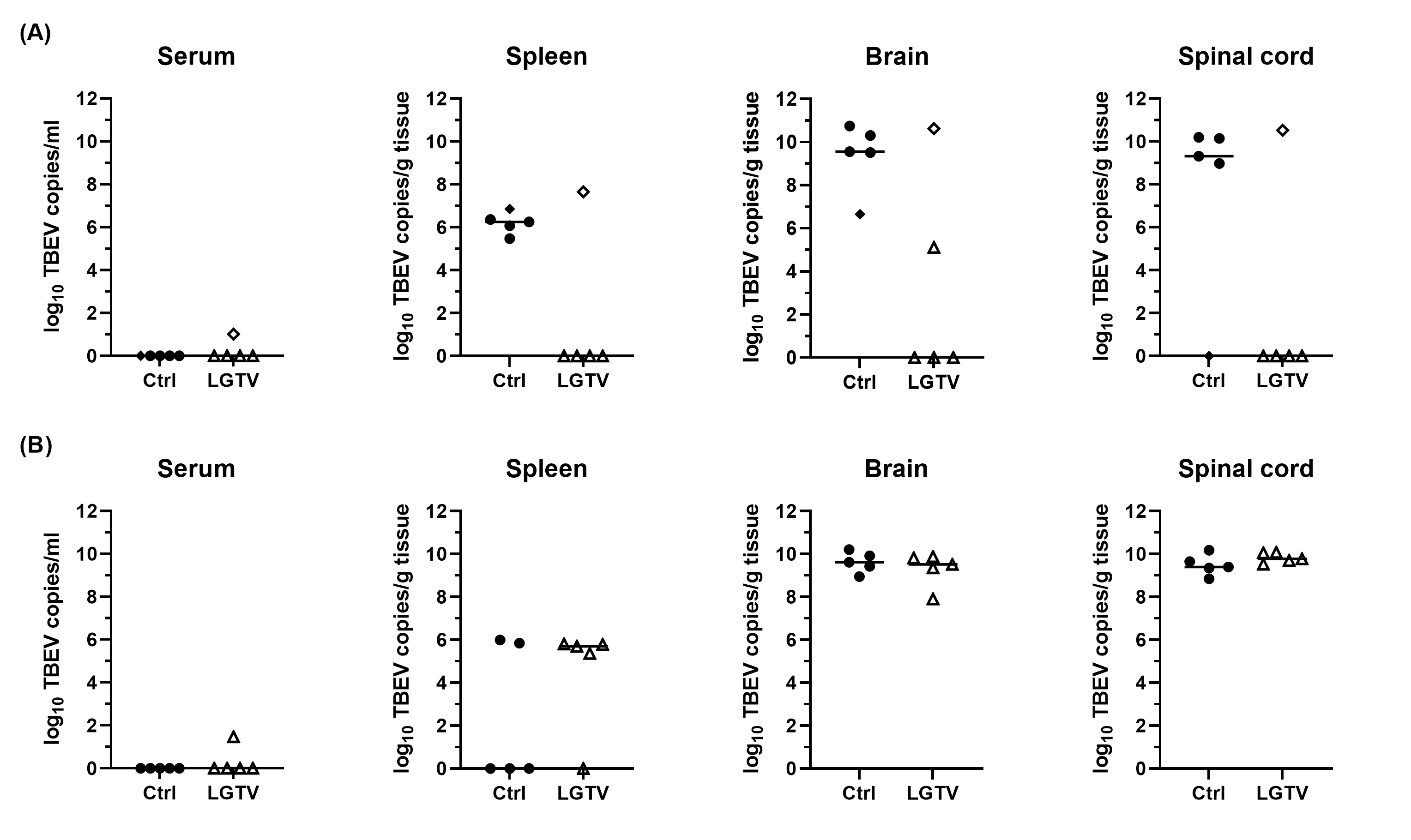

Supplement: Supplementary Figure 2 — Viral load in the organs of serum and T cell recipient mice that were challenged with TBEV. Real time quantitative RT-PCR was performed on total RNA isolated from serum and tissue homogenates collected at day of sacrifice of recipient mice which either received (A) serum or (B) CD3+ T cells from control (•) or LGTV (Δ) infected donor mice and were subsequently challenged with TBEV. The median is shown. Mice that differed in their clinical state from other mice in the control and LGTV serum recipient group are highlighted as rhombus shaped symbols [file Image_2.tif]

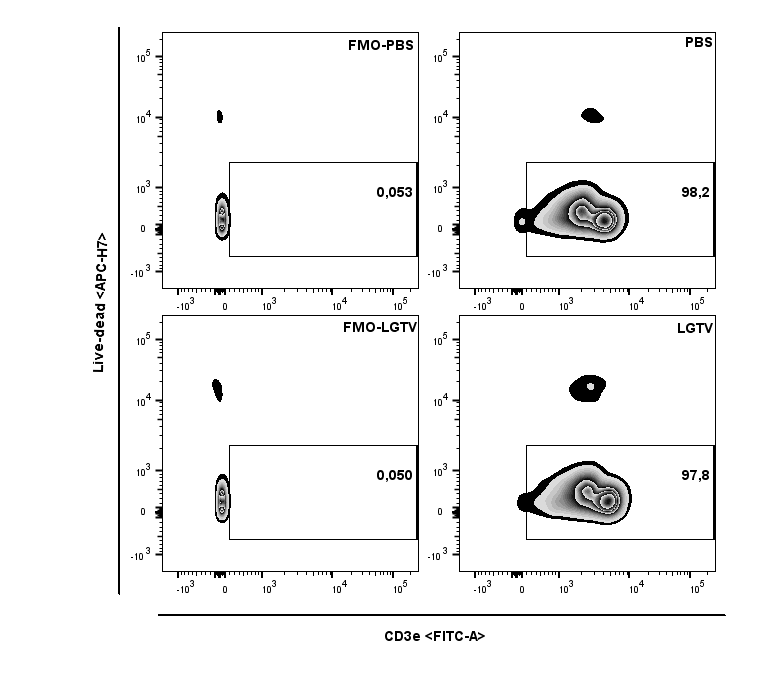

Supplement: Supplementary Figure 3 — Purity of adoptively transferred CD3+ T cell pools. Flow cytometric analysis of purified CD3+ T cells from control (upper panel) and LGTV (lower panel) donor groups prior to adoptive transfer to recipient mice. FACS plots are gated on live CD3+ T cells. [file Image_3.tiff]

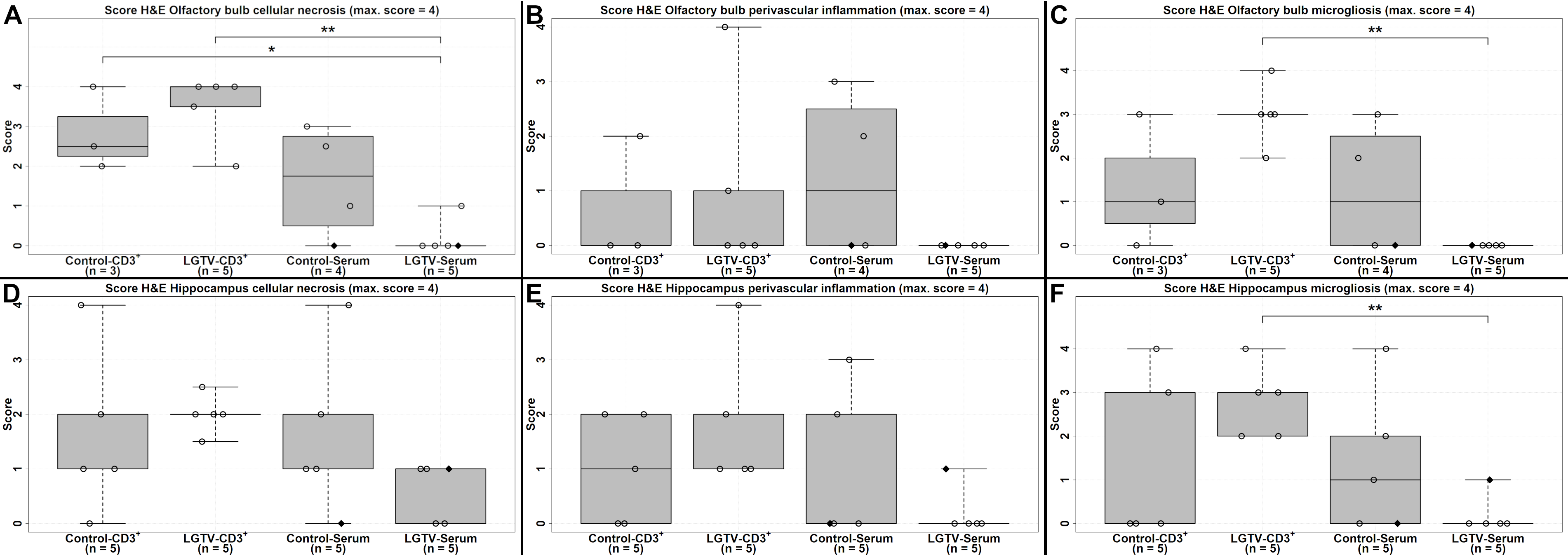

Supplement: Supplementary Figure 4 — Box plots of the hematoxylin and eosin (H&E) scoring values for “cellular necrosis” (A, D), “perivascular inflammation” (B, E) and “microgliosis” (C, F) of olfactory bulb (A-C) and hippocampus (D-F) for each experimental group. Significant differences detected by pairwise Wilcoxon rank-sum tests after non-parametric ANOVA are indicated by asterisks (* p < 0.05; ** p< 0.01; *** p < 0.001). Mice that differed in their clinical state from other mice in the control and LGTV serum recipient group are highlighted as rhombus shaped symbols in the box plots. [file Image_4.tif]

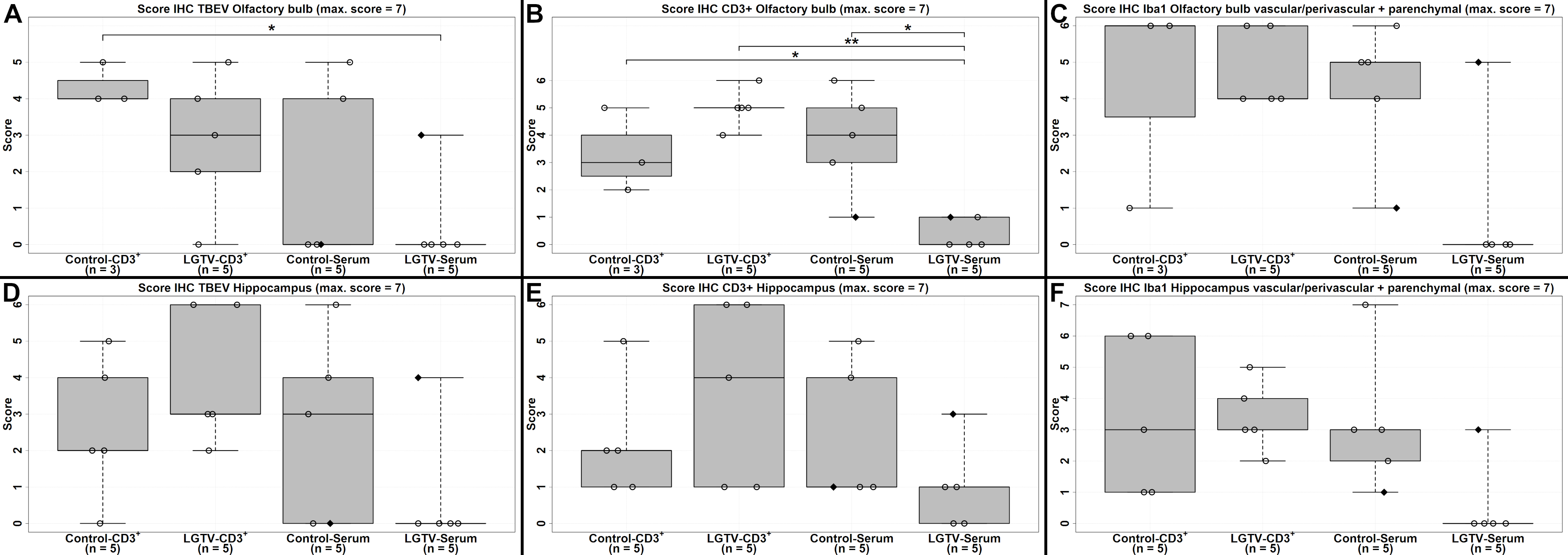

Supplement: Supplementary Figure 5 — Box plots of the scoring values of immunohistochemistry for TBEV (A, D), T cell marker CD3 (B, E) and microglia/macrophage marker Iba1 (C, F) of olfactory bulb (A-C) and hippocampus (D-F) for each experimental group. Significant differences detected by pairwise Wilcoxon rank-sum tests after non-parametric ANOVA are indicated by asterisks (* p < 0.05; ** p< 0.01; *** p < 0.001). Mice that differed in their clinical state from other mice in the control and LGTV serum recipient group are highlighted as rhombus shaped symbols in the box plots. [file Image_5.tif]

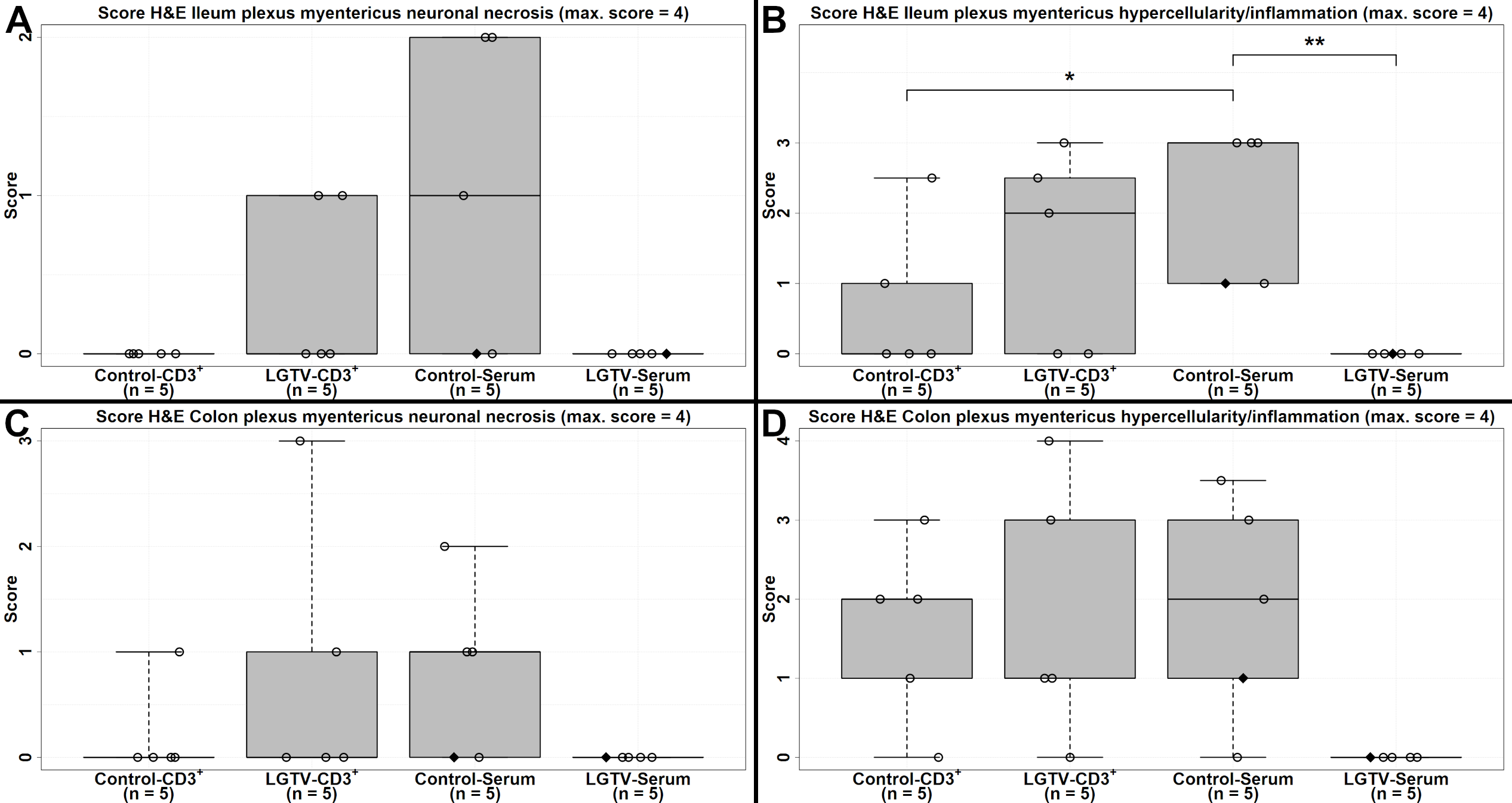

Supplement: Supplementary Figure 6 — Box plots of the hematoxylin and eosin (H&E) scoring values for “plexus myentericus neuronal necrosis” (A, C) and “plexus myentericus hypercellularity” (B, D) of ileum (A, B) and colon (C, D) for each experimental group. Significant differences detected by pairwise Wilcoxon rank-sum tests after non-parametric ANOVA are indicated by asterisks (* p < 0.05; ** p< 0.01; *** p < 0.001). Mice that differed in their clinical state from other mice in the control and LGTV serum recipient group are highlighted as rhombus shaped symbols in the box plots. [file Image_6.tif]

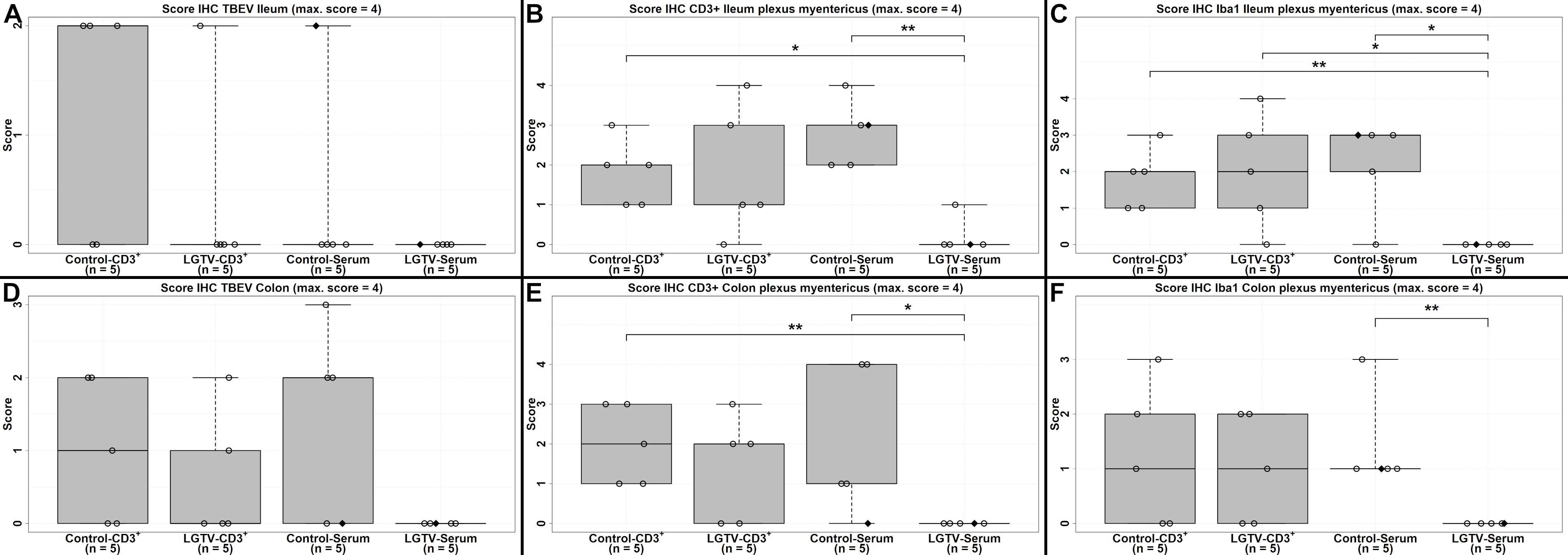

Supplement: Supplementary Figure 7 — Box plots of the scoring values of immunohistochemistry for TBEV (A, D), T cell marker CD3 (B, E) and microglia/macrophage marker Iba1 (C, F) of ileum (A-C) and colon (D-F) for each experimental group. Significant differences detected by pairwise Wilcoxon rank-sum tests after non-parametric ANOVA are indicated by asterisks (* p < 0.05; ** p< 0.01; *** p < 0.001). Mice that differed in their clinical state from other mice in the control and LGTV serum recipient group are highlighted as rhombus shaped symbols in the box plots. [file Image_7.tif]
